# Microglial depletion and repopulation in brain slice culture normalizes sensitized proinflammatory signaling

**DOI:** 10.1186/s12974-019-1678-y

**Published:** 2020-01-18

**Authors:** Leon G. Coleman, Jian Zou, Fulton T. Crews

**Affiliations:** 1 Bowles Center for Alcohol Studies, The University of North Carolina at Chapel Hill, School of Medicine, CB#7178, 1021 Thurston-Bowles Building, Chapel Hill, NC USA; 2 Department of Pharmacology, The University of North Carolina at Chapel Hill, School of Medicine, Chapel Hill, Chapel Hill, NC USA; 3 Department of Psychiatry, The University of North Carolina School of Medicine, Chapel Hill, Chapel Hill, NC USA

**Keywords:** Microglia, CSF1R, Microglial depletion, Microglial repopulation, Toll-like receptors, Ethanol, Priming

## Abstract

**Background:**

Microglia are critical mediators of neuroimmune pathology across multiple neurologic disorders. Microglia can be persistently activated or “primed” by Toll-like receptor (TLR) activation, ethanol, stress, and other insults. Thus, strategies to prevent or reverse microglial priming may be beneficial for conditions that involve progressively increasing microglial activation. Microglial depletion with repopulation is emerging as a potential therapy to normalize chronic immune activation. Primary organotypic hippocampal slice culture (OHSC) allows for the study of neuroimmune activation as well as microglial depletion and repopulation without involvement of peripheral immune activation. OHSC undergoes functional maturation and retains cytoarchitecture similar to *in vivo.*

**Methods:**

OHSC underwent microglial depletion with the CSF1R antagonist PLX3397 with or without repopulation after removal of PLX3397. Immune, trophic, and synaptic gene changes in response to agonists of TLRs 2, 3, 4, 7, and 9 as well as ethanol were assessed in the settings of microglial depletion and repopulation. Gi-DREADD inhibition of microglia was used to confirm select findings seen with depletion. The ability of microglial repopulation to prevent progressive proinflammatory gene induction by chronic ethanol was also investigated.

**Results:**

Microglia were depleted (> 90%) by PLX3397 in OHSC. Microglial depletion blunted proinflammatory responses to several TLR agonists as well as ethanol, which was mimicked by Gi-DREADD inhibition of OHSC microglia. Removal of PLX3397 was followed by complete repopulation of microglia. OHSCs with repopulated microglia showed increased baseline expression of anti-inflammatory cytokines (e.g., IL-10), microglial inhibitory signals (e.g., CX3CL1), and growth factors (e.g., BDNF). This was associated with blunted induction (~ 50%) of TNFα and IL-1β in response to agonists to TLR4 and TLR7. Further, chronic cycled ethanol from 4 days in vitro (DIV) to 16DIV caused immediate 2-fold inductions of TNFα and IL-1β that grew to ~4-fold of age-matched control slices by 40DIV. This persistent inflammatory gene expression was completely reversed by microglial depletion and repopulation after chronic ethanol.

**Conclusions:**

Microglia in OHSCs mediate proinflammatory responses to TLR agonists and ethanol. Microglial repopulation promoted an anti-inflammatory, trophic neuroenvironment and normalized proinflammatory gene expression. This supports the possibility of microglial depletion with repopulation as a strategy to reverse chronic neuroimmune activation.

## Background

Microglia are long-lived cells in the brain that respond to environmental sensitization. Microglial priming or sensitization to proinflammatory signaling has been associated with the progression of neurodegenerative diseases as well as normal aging [1–3], with “memory”-like adaptation of innate immune microglial signaling [4]. Microglial priming and phenotypes are poorly understood but are related to signaling of proinflammatory cytokines and Toll-like receptors (TLRs) [5]. The local neuroenvironment may also dictate microglial phenotype and result in unique microglia within and across brain regions that take on different functions [6, 7]. Microglial priming of proinflammatory signaling is increasingly being linked to depression, drug and alcohol dependence, brain stress responses, and neurological diseases [3, 8–11]. For these reasons, the inhibition of microglia has been explored as a therapeutic approach for multiple neurologic disorders. Interestingly, microglial depletion studies have found neuroprotective effects in multiple disease models [12–14], as well as reduced brain proinflammatory TNFα and IL1β responses to systemic lipopolysaccharide (LPS) [15] and acute binge ethanol [16]. Microglial depletion is a novel approach that can be used to provide insight into microglial biology and may have therapeutic potential in many brain diseases that involve neuroimmune dysfunction [17, 18].

Microglia can be depleted from the brain by both genetic and pharmacological approaches [19]. Microglia depend upon constant signaling through the colony-stimulating factor 1 receptor (CSF1R) for survival. CSF1R antagonists, e.g., PLX3397, successfully deplete up to 99% of microglia in vivo [20, 21]. Surprisingly, most studies have found little or no behavioral consequences of microglial depletion, although, in models of various diseases, microglial depletion by CSF1R antagonists has varying outcomes [18]. For example, CSF1R inhibition has been found to be neuroprotective in the contexts of intracerebral hemorrhage [12] and diphtheria toxin-induced neuronal injury [13]. However, CSF1R inhibition was associated with more severe brain injury and neuroimmune activation in ischemia models [22, 23]. Thus, the benefit or detriment of microglial depletion varies across different disease states. Depleted microglia are able to repopulate *in vivo*, though there has been debate regarding the origin of repopulated microglia [7, 23–27]. Early reports suggested proliferation of microglia from neuroprogenitors [25], though subsequent fate mapping studies found repopulation from remaining microglia [26]. Others find infiltration of peripheral macrophages contributes to new microglia [7, 23, 27], and a recent report from Lund et al. found microglia repopulated both from local proliferation of remaining microglia and infiltration of peripheral macrophages, with each population resulting in distinct microglial activation profiles [7]. Thus, responses of repopulated microglia *in vivo* may be complicated by differential response patterns of microglia from different origins. Organotypic hippocampal slice cultures (OHSC) are an ex vivo model of in vivo brain without peripheral confounds. OHSC has all brain cell types in the normal architecture of the brain [28], survives for long periods [29], and has functional maturation of synapses [30–33]. OHSCs have been used to effectively model microglial regulation of excitatory synapses [34], microglial remodeling of synapses [35], microglial protection of neurons during excitoxicity and ischemia [36, 37], and microglial depletion and replenishment with microglial transplants [34, 37, 38]. We have previously used OHSCs to study alcohol-induced neuroinflammation and TLR activation [39, 40]. Thus, OHSC in large part mimics in vivo brain without the confounders of systemic immune signals [28]. Therefore, we used OHSC to investigate microglial priming, TLR signaling, and the impact of microglial depletion and repopulation on the consequences of immune signaling without systemic influences*.*

TLR expression in the brain is increased in several neurologic diseases including Alzheimer’s disease, depression, and alcohol use disorders (AUDs). TLRs are expressed in microglia, astrocytes, and neurons with different functional consequences in each cell type and likely contribute to microglial priming. We have found increased expression of TLRs 2–4, 7, and 9 as well as microglial markers in brain with alcohol (i.e., ethanol) [39, 41–45]. TLR signaling is linked to microglial priming, sensitization, and progressive worsening of many pathologies [9, 46, 47]. We report here that agonists to TLRs 2,3,4,7, and 9 agonists alter proinflammatory cytokines and synaptic markers in OHSCs. We found PLX3397 treatment of OHSC results in significant microglial depletion and large reductions in TLRs 2, 3, 4, 7, and 9 agonist-induced gene induction and proinflammatory immune responses. Similarly, microglia depletion with PLX3397 also prevents ethanol-mediated proinflammatory gene induction. In addition to the effects on microglial genes, we also assessed effects of microglial depletion and repopulation on expression of genes associated with neurons and astrocytes to gain an overall picture of the neuroenvironment. Removal of PLX3397 from culture media results in progressive microglial repopulation similar to findings in vivo [25]. Interestingly, we find OHSC with endogenously repopulated microglia has decreased responses to TLR agonists, increased synaptic and trophic factor gene expression, and loss of proinflammatory priming, as well as altered microglial, neuronal, and astrocyte gene expression. Further, microglial depletion with repopulation afterward, reversed enhancement of proinflammatory gene expression following chronic ethanol. These studies support OHSC as a model that maintains the microglial environmental niche and finds that PLX3397 microglial depletion and repopulation results in a microglial phenotype with reduced proinflammatory TNFα and IL1β responses.

## Methods

### Overall experimental design

**Fig. 1:**
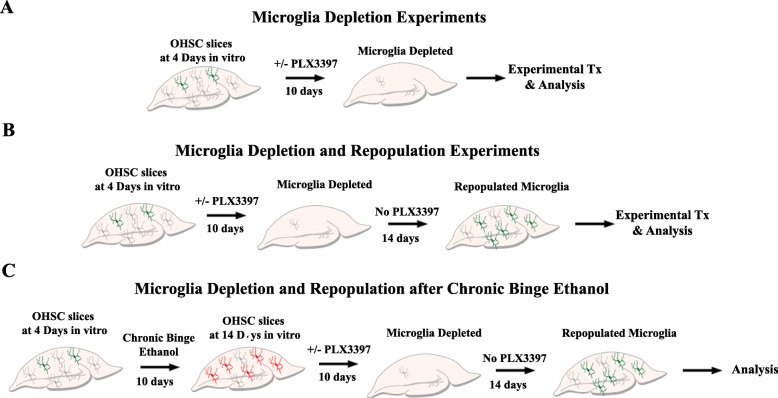
Overall experimental design. **a** Experimental design for microglial depletion *ex vivo.* OHSCs were prepared as described below. On day 4 in vitro (4DIV), slices were treated with PLX3397 (1 μM) for 10 days to deplete microglia and then removed for analysis. **b** Protocol of microglial depletion and repopulation. OHSCs at 4DIV were treated with PLX3397 (1 μM) for 10 days and then returned to PLX-free medium for different time points as indicated. **c** Experimental design for microglial repopulation after chronic ethanol. OHSCs were treated with chronic binge ethanol (2 days on, 2 days off for 10 days) followed by microglial depletion and repopulation. Slices were analyzed after 14 days of repopulation

The experimental protocols were performed as described in Fig. 1. In one set of experiments, rat organotypic brain slice cultures underwent microglial depletion with or without treatment with ethanol or other TLR agonists (Fig. 1a). In the second set of experiments, slices underwent microglial depletion with microglial repopulation (Fig. 1b). In a third set of experiments, microglia depletion and repopulation occurred after chronic binge ethanol exposure, to assess for a potential reversal of persistent ethanol effects (Fig. 1c).

### Organotypic hippocampal slice culture (OHSC)

Rat organotypic hippocampal-entorhinal cortical slice cultures were prepared as described previously [48, 49]. Briefly, neonates rat pups at postnatal day 7 (P7) were decapitated, brain removed and hippocampal-entorhinal complex dissected in Gey’s buffer (Sigma-Aldrich, St. Louis, MO). Slices were transversely cut with McIlwain tissue chopper at a thickness of 375 μm and placed onto a 30-mm-diameter membrane tissue insert, 10–13 slices per tissue insert. Slices were cultured with medium containing 75% glutamate-free MEM with 25 mM HEPES and Hank’s salts +25% horse serum (HS) + 5.5 g/L glucose + 2 mM l-glutamine in a humidified 5% CO2 incubator at 36.5 ^°^C prior to and during depletion. OHSCs survive for weeks and maturation continues ex vivo with neuronal synaptic maturation [29] and levels of dentate gyrus neurogenesis reaching levels found in rat adolescent hippocampus [50]. All protocols followed in this study were approved by the Institutional Animal Care Use Committee of The University of North Carolina at Chapel Hill and were in accordance with National Institutes of Health regulation for the care and use of animals in research.

### CSF1R antagonism, TLR, and ethanol treatments

OHSC microglial depletion and repopulation experiments followed an initial 4 days in vitro (DIV). OHSC slices at 4DIV were treated with PLX3397 (1 μM) for 10 days to deplete microglia. TLR agonist responses were determined after this 10-day PLX3397 treatment as indicated in the results. In the last 4 days of PLX3397, serum was reduced to 12% in media to gradually reduce serum prior to repopulation. During microglial repopulation, slices were maintained in N2-supplemented MEM containing 6% HS and removed at indicated time points for further analysis. To determine TLR agonist responses, slices were treated with various TLR agonists at the indicated concentrations for 16 h, both immediately after PLX3397 microglial depletion and after 14 days of continued culture microglial repopulation. The following TLR agonists were purchased from InvivoGen (San Diego, CA): Pam3CSK4 (TLR2), PolyI:C (TLR3), Lipopolysaccharide/LPS (TLR4), Imiquimod/IMQ (TLR7), and D-SL03 (TLR9). Ethanol treatment with the indicated concentrations occurred in a desiccator containing 300 ml water saturated with equal concentrations of ethanol to balance evaporation of ethanol from the media. At the end of experiments, slices were removed for further analysis. For binge ethanol exposure, OHSC slices at 4DIV were exposed to ethanol (100 mM) for 3 cycles in which cultures were cycled in ethanol-containing medium for 2 days and in ethanol-free medium for 2 days (2 days on/ 2 days off); at the beginning of the next ethanol exposure, ethanol concentration in culture medium was measured and replenished to 100 mM. At the end of the binge ethanol exposure (total duration 12 days), slices underwent microglial depletion and followed by microglial repopulation. Slices were removed at indicated time points for further analysis.

### Gi-inhibitor Designer Receptor Exclusively Activated by a Designer Drug (DREADD) chemogenetic inhibition of microglia

OHSCs were for 24 h incubated with an AAV9-CD68-hM4Di-mCherry viral vector to selective express the inhibitory Gi-DREADD in microglia. This vector has been validated previously in rats in vivo [51, 52]. Specific viral expression in microglia was confirmed by immunofluorescence. Slices were then treated with either IMQ or ethanol +/− the ligand for hM4Di, CNO (0–1 μM).

### 5-Bromo-2′-deoxyuridine (BrdU) labeling, immunohistochemistry and ELISAs

OHSC slices undergoing microglial depletion (10-day PLX3397 treatment) were treated with BrdU (50 μM, Sigma-Aldrich) on day 9 of PLX3397 treatment for 24 h to label repopulating cells. OHSC slices in BrdU-free, PLX3397-free culture medium containing 6% HS were removed at different time points for analysis. For BrdU immunohistochemistry, the slices were removed and fixed with 4% paraformaldehyde +5% sucrose in 0.1 M PBS overnight at 4 °C and stained for BrdU uptake with the modified method of [53]. Briefly, the whole slices were detached from the tissue insert membrane, washed, and denatured with 2 N HCL for 60 min at 37 °C. The slices were then washed with 0.1 M boric acid buffer (pH 8.5) and blocked with HS for 60 min at room temperature. The slices were incubated with mouse anti-BrdU antibody (1:1000, Chemicon, Temecula, CA) in PBS containing 3% HS + 0.25% Trion X-100 for 48 h. The slices were processed for avidin-biotin-peroxidase reaction as described elsewhere [54], dehydrated, mounted, and visualized with microscopy. ELISAs for media protein levels of HMGB1 (IBL, International), IL-4 (ThermoFisher), and BDNF (RayBioTech) were performed according to the manufacturer’s instructions. Media glutamate levels from each experiments were determined with Glutamate ELISA kit (Abnova, Taipei City, Cat#:KA1909). Briefly, a total of 100 μl of culture medium from each sample was used. After extraction and derivatization, glutamate was quantitatively determined by ELISA according the manufacturer’s instruction. All samples were run in duplicate.

### Double immunofluorescence

Free-floating slices were processed for double immunofluorescence staining. The primary antibodies used were as follows: mouse anti-BrdU (1:1000) and Iba-1 (1:1000, Wako, Richmond, VA) for microglia. Alexa Fluor 594 anti-rabbit and Alexa Fluor 488 anti-mouse IgG (1:1000, Invitrogen, Carlsbad, CA) were secondary. All slices were incubated with primary antibody for 48 h at 4 °C and 2 h with secondary antibody at room temperature. The slices were washed in PBS, mounted and coverslipped.

### Microparticle (MP) isolations and miRNA analysis

Culture media was collected at the end of experiments as indicated and centrifuged at 6000*g* for 10 min to remove cellular debris. The supernatant was then collected and centrifuged at 21000*g* for 96 min at 4 °C to isolate microparticles (MP) as describe previously [43, 55]. For miRNA analysis, pelleted MPs total RNA was obtained using miRNeasy Kit (Qiagen Inc., CA) according to the manufacturer’s protocol. The assessment of miRNAs was performed by standard TaqMan miRNA Assays as described previously [43]. The expression of miRNA levels were normalized to snU6.

### RNA isolation, reverse transcription, and real-time quantitative RT-PCR

For each specific experiment, the slices were removed at the end of experiment and rinsed with cold PBS, followed by total RNA purification using miRNeasy Kit (Qiagen, CA). The total amount of RNA was quantified by nanodrop. For reverse transcription, 2 μg of RNA was used to synthesize the first strand of cDNA using random primers (Invitrogen) and reverse transcriptase Moloney murine leukemia virus (Invitrogen). After a 1:2 dilution with water, 2 μl of the first strand cDNA solution was used for RT-PCR. The primer sequences for real-time RT-PCR are shown in Additional file 1: Table S1. SYBR Green SuperMix (Applied Biosystems, UK) was used as a RT-PCR solution. The real-time RT-PCR was run with initial activation for 10 min at 95 °C and followed by 40 cycles of denaturation (95 °C, 40 s), annealing (58 °C, 45 s), and extension (72 °C, 40 s). The threshold cycle (*C*_T_) of each target product was determined and normalized to internal standard β-actin or 18S. The difference in *C*_T_ values (ΔΔ*C*_T_) of two genes was calculated $\left[\mathrm{difference}={2}^{-\left({\mathrm{C}}_{\mathrm{T}}\;\mathrm{of}\kern0.17em \mathrm{target}\kern0.17em \mathrm{genes}-{\mathrm{C}}_{\mathrm{T}}\;\mathrm{of}\;\upbeta -\mathrm{actin}\right)}={2}^{-{\mathrm{C}}_{\mathrm{T}}}\right]$, and the result was expressed as the percentage compared to control.

### Statistical analysis

Statistical comparisons were made with ANOVA, and the difference between the experimental groups was further compared by using post hoc Fisher PLSD test. Differences were considered to be statistically significant if *p* < 0.05. For all gene expression comparisons, treatment groups were compared to untreated controls (i.e., no microglial depletion or repopulation) that spent the same number of days in culture.

## Results

### Microglial depletion by PLX3397 in brain slice culture blunts TLR immune responses

**Fig. 2:**
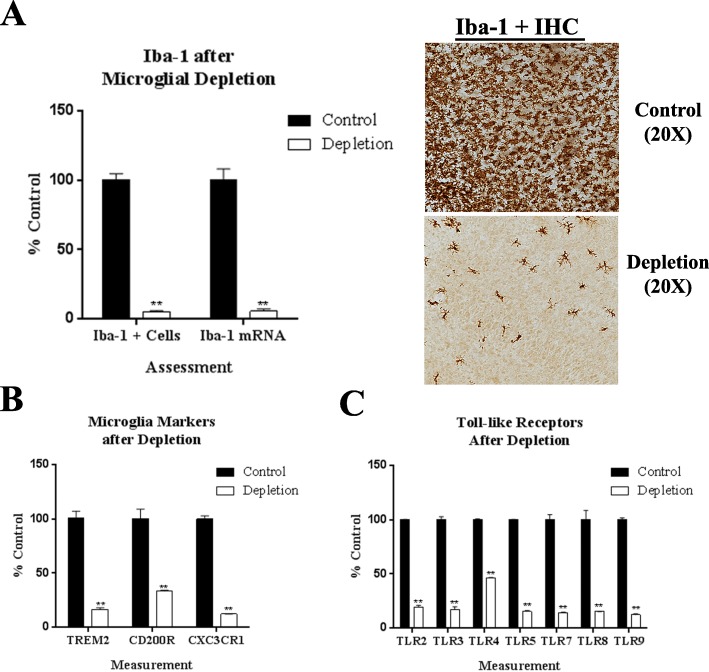
Microglial depletion by CSF1R inhibitor PLX3397 in primary ex vivo organotypic hippocampal slice culture (OHSC). **a** Quantification of microglial depletion in ex vivo OHSC by PLX3397A. Iba-1+ microglia count or Iba-1 mRNA in control and microglia depletion (M-Dep) groups. Representative Iba-1+ microglia immunostaining images are shown on the right. **b** Microglial marker gene expression was assessed by RT-PCR control and microglia depletion groups. **c** Gene expression of Toll-like receptors (TLRs) 2–9 following microglial depletion. *p < 0.05, **p < 0.01 vs control; *n* = 3 replicates/group

Emerging studies have suggested that microglial depletion by multiple methods has beneficial effects on pathology [17, 18]. CSF1R antagonists have been shown previously to effectively deplete microglia in vivo. OHSCs maintain the cellular structure of the hippocampus ex vivo [29], and we confirmed by RT-PCR that OHSC retain expression of Sall1 (not shown), a critical microglial transcriptional regulator lost in some microglial culture preparations [56]. We hypothesized that CSF1R antagonist PLX3397 would effectively deplete microglia in OHSC. As described in the methods, after 4 days in vitro (4DIV), OHSC slices were treated with PLX3397 (1 μM) for 10 days day (Fig. 1a). OHSC have many Iba-1+ cells and mRNA expression that is markedly reduced by PLX3397 treatment. PLX3397 reduced Iba-1+ cells and mRNA by more than 90% as assessed by immunohistochemistry (IHC) and RT-PCR, respectively (Fig. 2a). Further, gene expression of additional microglial markers, such as TREM2 (80% reduction), CD200R (65%), and CX3CR1 (90%), was substantially reduced (Fig. 2b). Microglia express high levels of immune pattern recognition receptors such as TLRs. Therefore, we assessed TLR expression levels after microglial depletion. Expression of TLRs 2–6, 7, and 9 were all reduced by at least 80%, with the exception of TLR4, which was reduced by 55% (Fig. 2c). Although most microglia were depleted by PLX3397 exposure, a subset remained, similar to in vivo studies [17]. Thus, OHSC express microglia, and similar to in vivo studies, microglia are effectively depleted by PLX3397 as indicated by reduced expression of Iba-1, TREM2, CX3CR1, and multiple TLRs.

**Fig. 3:**
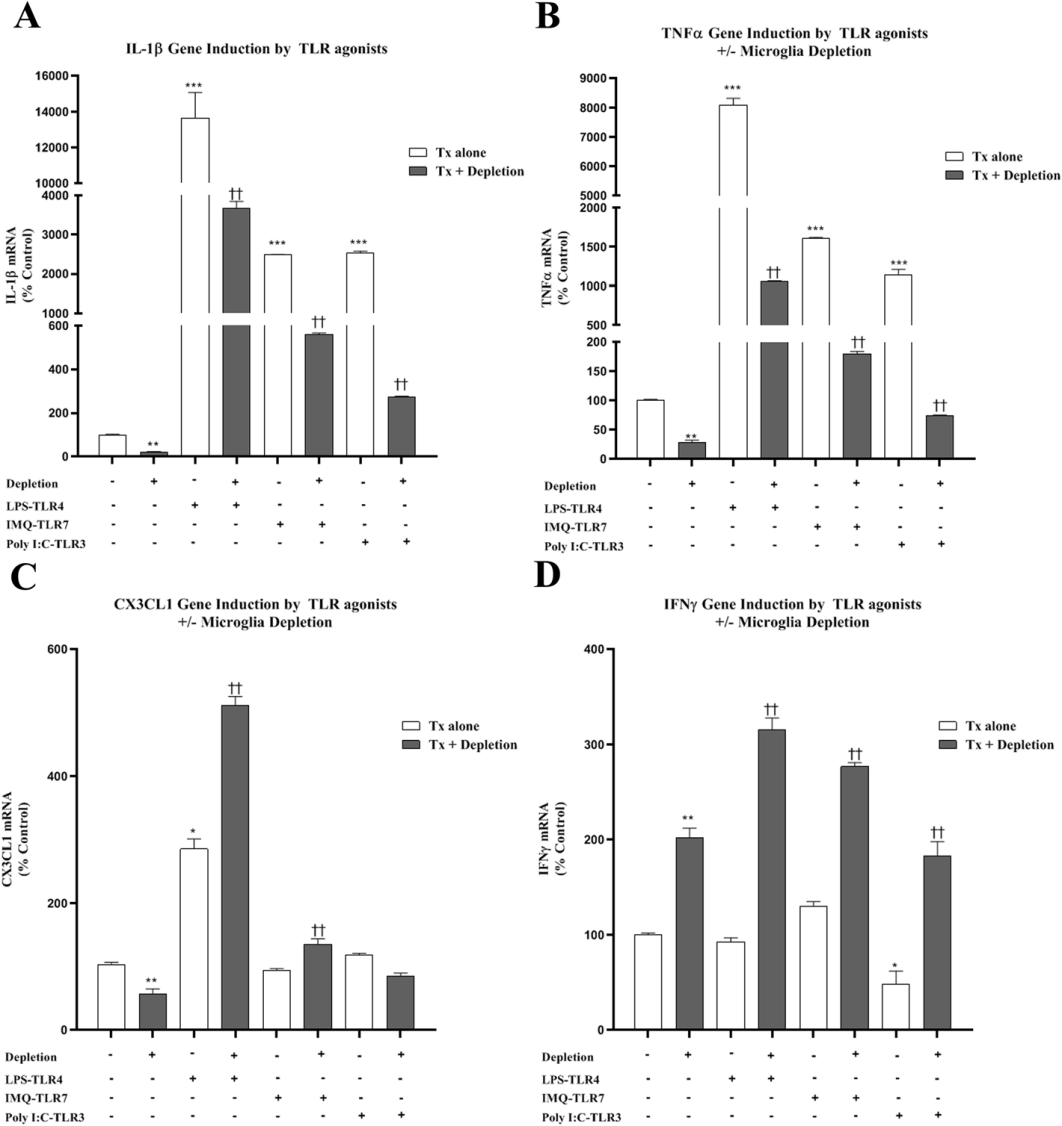
Microglial depletion blunts proinflammatory gene induction to TLR agonists. OHSC slices at 4DIV were treated with CSF1R inhibitor PLX3397 for 10 days to deplete microglia, followed by treatment of different TLR agonists for 16 h. Immune gene induction was assessed by RT-PCR. Microglial depletion reduced induction of **a** IL-1β and **b** TNFα in response to LPS (TLR4), IMQ (TLR7), and PolyI:C (TLR3). Microglial depletion either had no effect or enhanced induction of **c** CX3CL1 and **d** IFNγ. *p < 0.05, **p < 0.01 vs Control, †*p <* 0.05 vs respective TLR agonist; *n* = 3 replicates/group with at least two independent experiments per condition

Multiple brain cells express TLR receptors, although microglia are known TLR-responding cells. We assessed OHSC proinflammatory mRNA in controls as well as following microglial depletion and immune responses to known TLR agonists, e.g., TLR2 (Pam3CSK4, Pam3C), TLR3 (PolyI:C), TLR4 (LPS), TLR7 (Imiquimod, IMQ), and TLR9 (D-SL03) (Table 1, Fig. 3). TLR responses were assessed at 16 h, a time point we have found shows robust induction of TNFα and IL-1β with TLR agonists (unpublished observations). Microglial depletion markedly reduced OHSC mRNA for TNFα and IL1β as well as CX3CR1 and CD200R. Interestingly, interferon alpha (IFNα) and interferon gamma (IFNγ) were not reduced. Agonists to TLRs 2, 3, 4, 7, and 9 increased proinflammatory cytokines TNFα and IL-1β mRNA from between 10- and over 200-fold, whereas IFNγ mRNA was increased 2- to 3-fold (Fig. 3). PLX3397 microglial depletion markedly reduced proinflammatory responses. For example, LPS caused a 225-fold increase in IL-1β mRNA that was reduced by 84% microglial with depletion (Fig. 3a). Similarly, the TLR7 agonist IMQ and the TLR3 agonist PolyI:C caused about a 25-fold induction of IL-1β mRNA that was markedly blunted by microglial depletion. TNFα mRNA responses were also reduced by microglial depletion: TLR4/LPS (87%), TLR7/IMQ (89%), and TLR3/PolyI:C (94%). This is consistent with TNFα and IL-1β induction by TLR agonists in slice culture occurring primarily in microglia. To confirm the inhibition of TNFα induction was due to the loss of microglial activity, we used a microglial specific Gi-inhibitory DREADD (AAV9-CD68-hM4Di-mCherry) to selectively inhibit microglial function as described previously [51, 52]. We observed successful transfection of the hM4Di-mCherry construct specifically in microglia (Additional file 3: Figure S1A). Inhibition of hM4Di-transfected microglia in OHSC with the addition of CNO inhibited TLR7-mediated induction of TNFα in a concentration-dependent fashion (Additional file 3: Figure S1B), consistent with our findings with microglial depletion. Type I and type II interferons, however, showed different response patterns. IFNα gene expression (type I) was increased by each of the TLR agonists with corresponding reduction by microglial depletion (Table 1), whereas IFNγ (type II) expression was enhanced by microglial depletion, suggesting IFNγ expression occurs in other cell types and may be suppressed by microglia (Fig. 3c). Both microglial depletion and proinflammatory activation of microglia reduced the fractalkine receptor mRNA (CX3CR1). Fractalkine ligand expression mRNA (CX3CL1), which is neuronal, showed varied responses, being induced by agonists to TLRs 2, 4, and 9. Microglial depletion caused enhanced CX3CL1 mRNA responses to agonists of TLRs 2, 4, 7, and 9 suggesting neuronal induction of fractalkine by TLR agonists in the absence of microglia. These findings indicate PLX3397 microglial depletion in OHSC markedly reduces TLR pro-inflammatory gene induction.

**Table 1:** Depletion of microglia reduces immune activation in response to TLR agonists. Brain slices were treated with vehicle or underwent microglial depletion with PLX3397 for 10 days, followed by treatment with a TLR agonist. Gene induction was assessed by RT-PCR at 16 h. Mean ± SEM

Treatment +/− PLX3397	Inflammatory cytokines		Interferons		CD200-CD200R Axis		CX3CL1-CX3CR1 axis	
	TNFα	IL-1β	IFNα	IFNϒ	CD200	CD200R	CX3CL1	CX3CR1
Control	100 ± 2	100 ± 3	102 ± 1	100 ± 2	98 ± 6	101 ± 16	103 ± 4	98 ± 4
+PLX3397	29 ± 3^**^	21 ± 2**	118 ± 18	202 ± 10**	64 ± 8*	21 ± 0.4**	57 ± 8**	20 ± 3**
Pam3C (TLR2)	9165 ± 603*	27429 ± 2181*	140 ± 2*	74 ± 11*	86 ± 6	71 ± 2*	265 ± 14*	15 ± 1*
+PLX3397	746 ± 10^††^	3128 ± 162^††^	81 ± 6^††^	104 ± 1	52 ± 1	18 ± 0.3^††^	455 ± 11^††^	3 ± 0.1^††^
PolyI:C (TLR3)	1145 ± 67*	2534 ± 44*	132 ± 7*	48 ± 14*	52 ± 4*	39 ± 0.5*	118 ± 3	55±
+PLX3397	74 ± 1^††^	275 ± 2^††^	84 ± 1^††^	183 ± 15^††^	68 ± 3	7 ± 0.5^††^	85 ± 5	8 ± 0.4^††^
LPS (TLR4)	8104 ± 225*	22460 ± 2788*	399 ± 21*	93 ± 4	80 ± 2	39 ± 1*	286 ± 15*	8 ± 0.4*
+PLX3397	1056 ± 11^††^	3667 ± 178^††^	106 ± 7^††^	316 ± 12^††^	94 ± 12	9 ± 0.5^††^	512 ± 14^††^	1 ± 0.02^††^
IMQ (TLR7)	1606 ± 17*	2485 ± 9*	188 ± 4*	130 ± 5	81 ± 2	42 ± 3*	94 ± 3	34 ± 1*
+PLX3397	180 ± 4^††^	560 ± 6^††^	264 ± 7^††^	277 ± 4^††^	141 ± 26^††^	7 ± 0.3^††^	135 ± 9^††^	10 ± 1^††^
D-SL03 (TLR9)	9639 ± 334*	7211 ± 399*	209 ± 19*	165 ± 26*	92 ± 4	12 ± 0.1*	249 ± 15*	23 ± 0.1*
+PLX3397	1234 ± 21^††^	1151 ± 4^††^	151 ± 16^††^	287 ± 10^††^	79 ± 4	1 ± 0.01^††^	209 ± 9	5 ± 0.1^††^

*p < 0.05, **p < 0.01 vs control; †*p* < 0.05 vs corresponding TLR agonist control, ††*p* < 0.01 vs corresponding TLR agonist

### Microglial depletion by PLX3397 blunts immune responses to ethanol

**Fig. 4:**
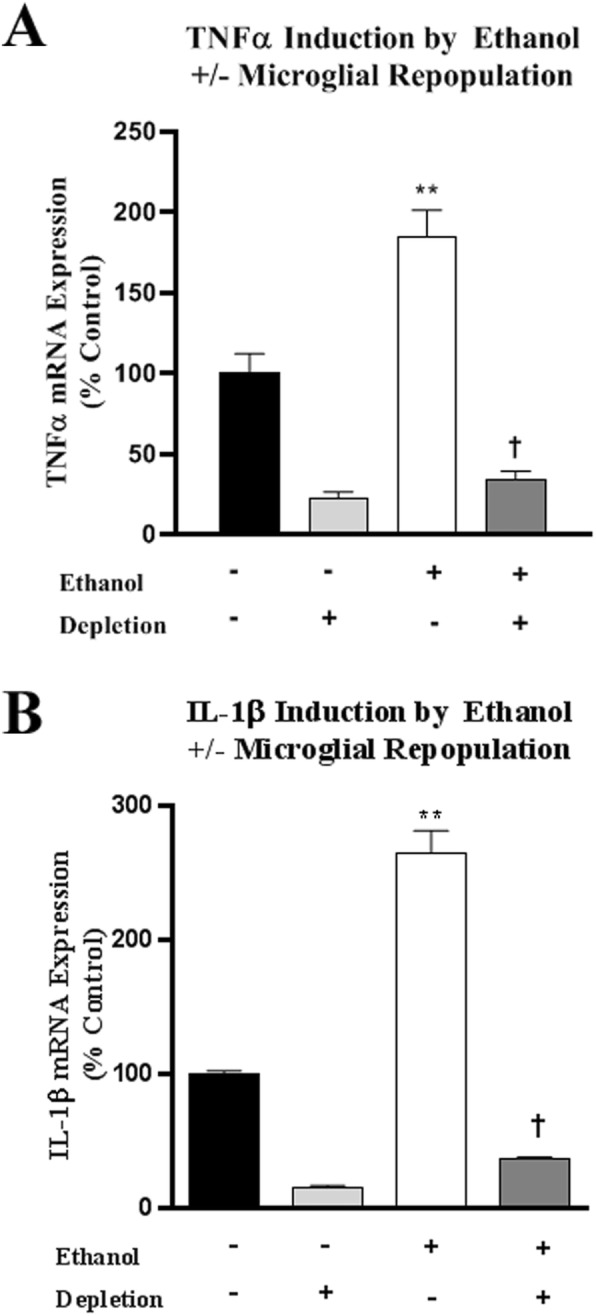
Microglial depletion blunts induction of proinflammatory genes by ethanol. OHSCs at 4DIV were treated with CSF1R inhibitor PLX3397 for 10 days to deplete microglia and followed by treatment of ethanol (100 mM, 4 days). At the end of ethanol treatment, slices were removed for RT-PCR analysis of proinflammatory immune gene induction. Ethanol significantly induced **a** TNFα (1.8-fold) and **b** IL-1β (2.8-fold). Both of these responses were abolished in microglial depletion.**p < 0.01 vs control, †*p <* 0.05 vs. ethanol. *N* = 3 replicates with at least three independent experiments per condition

Emerging preclinical and human brain studies find microglial sensitization and TLR induction contribute to the development of alcohol use disorder (AUD) and AUD-associated neuropathology [43, 57–60]. Ethanol treatment of OHSC has been found to increase expression of multiple proinflammatory cytokines related to increases in NFκB p65 phosphorylation and binding to DNA [40, 43, 49]. Previous studies have found PLX3397 treatment in mice alters acute ethanol-mediated brain proinflammatory responses [16]. Control vehicle or PLX3397 microglia-depleted OHSC was exposed to a binge drinking ethanol concentration (Fig. 1a, 100 mM). Multiple immune and neuronal signaling genes were assessed at 4 days; a chronic treatment we have found previously induces proinflammatory gene expression [43, 50, 59]. Similar to in vivo studies, ethanol exposure increased mRNA for proinflammatory genes TNFα (1.9-fold) and IL-1β (2.6-fold) gene expression, which was eliminated by microglial depletion (Fig. 4a, b). To confirm the role of microglia in TNFα induction by ethanol, we used Gi-DREADD inhibition as above. Inhibition of hM4Di-transfected microglia in OHSC with the addition of CNO inhibited ethanol induction of TNFα in a concentration-dependent fashion (Additional file 3: Figure S1C), consistent with our findings following microglial depletion. Interestingly, ethanol exposure increased IL-6 and IL-10 mRNA, which were not blunted by microglial depletion, suggesting ethanol induction in other brain cell types (Table 2). Ethanol exposure increased Iba-1 and most TLR mRNA, which were generally reduced by microglial depletion with reduced microglial marker gene expression. Previous studies have found ethanol increases proinflammatory miRNA in microglia and macrophages [43, 61], prompting assessments in OHSC and in media to follow secretion. Ethanol exposure increased miRNA Let-7b, miR155, miR21, and miR-181 in both slices and media microparticles (Additional file 4: Figure S2). In microglia-depleted slices, ethanol induction and release of miR-155, miR-21, and miR-181 were markedly reduced, suggesting these miRNA are involved in the microglial response to ethanol (Fig. 4). Ethanol also increased mRNA for glutamate receptor subunits NR2A and mGluR8, as well as the trophic factor BDNF. Interestingly, these genes were further induced in the setting of microglial depletion and ethanol, suggesting their induction by ethanol is microglia-independent, and may actually be inhibited by the presence of microglia (Table 2). Similarly, IFNα induction by ethanol was also further increased after microglia depletion. These findings indicate that ethanol-induced microglial signaling impacts the expression of trophic, synaptic, miRNA and IFN innate immune signaling genes in other brain cells. Further, ethanol induction of TNFα, IL-1β, TLRs, and pro-inflammatory miRNAs require microglia, while ethanol induction of IL-10, IL-6, and IFNα does not. Therefore, either depletion or inhibition of microglia may prevent the majority of pro-inflammatory responses to ethanol.

**Table 2:** Depletion of microglia reduces immune activation in response to ethanol. Brain slices were treated with vehicle or underwent microglial depletion with PLX3397 for 10 days, followed by ethanol (100 mM) treatment without PLX3397. Gene induction was assessed by RT-PCR after 4 days of ethanol treatment. Mean ± SEM

Category	Gene	Control	Depletion	Ethanol	EtOH + depletion
Cytokines	TNFα	100 ± 12	22 ± 4**	185 ± 16**	34 ± 5^††^
	IL-1β	100 ± 2	15 ± 2**	264 ± 17**	37 ± 1^††^
	IL-4	100 ± 14	239 ± 38*	34 ± 6*	139 ± 11^††^
	IL-6	100 ± 14	356 ± 20**	186 ± 50*	335 ± 6^††^
	IL-10	100 ± 4	151 ± 10*	217 ± 3**	224 ± 18^††^
Toll-like receptors	TLR2	100 ± 0.4	56 ± 0.2**	184 ± 25**	68 ± 10^††^
	TLR3	100 ± 2	55 ± 6**	235 ± 3**	101 ± 2^††^
	TLR4	100 ± 3	104 ± 14	159 ± 1*	113 ± 6
	TLR5	100 ± 5	16 ± 2**	112 ± 9	30 ± 2
	TLR7	100 ± 9	23 ± 1**	195 ± 5**	49 ± 0.4^††^
	TLR9	100 ± 8	24 ± 1**	189 ± 5**	48 ± 9^††^
Microglia markers	Iba-1	100 ± 0.3	21 ± 1**	149 ± 5*	45 ± 3^†^
	TREM2	100 ± 9	14 ± 0.5	76 ± 13*	32 ± 1^††^
	CD200R	100 ± 9	33 ± 1**	78 ± 4*	50 ± 5^††^
	CX3CR1	100 ± 3	12 ± 0.1**	69 ± 1*	116 ± 4
	IL-4R	100 ± 10	37 ± 4**	144 ± 20*	66 ± 9^††^
	IRF8	100 ± 2	15 ± 0.1**	66 ± 3*	15 ± 1^††^
Neuronal genes	mGluR2	100 ± 6	263 ± 56**	134 ± 11	158 ± 18^†^
	mGluR3	100 ± 9	170 ± 11*	92 ± 6	206 ± 24
	mGluR5	100 ± 2	173 ± 23*	94 ± 21	167 ± 15^†^
	mGluR8	100 ± 10	213 ± 38**	169 ± 4*	364 ± 6^††^
	NR2A	100 ± 1	133 ± 0.5**	135 ± 5*	272 ± 7^††^
	NR2B	100 ± 5	137 ± 1*	123 ± 7	264 ± 2^††^
	PSD-95	100 ± 2	153 ± 21*	124 ± 1	201 ± 58^†^
	BDNF	100 ± 9	115 ± 6	205 ± 8**	550 ± 4^††^
	NGF	100 ± 3	115 ± 7	148 ± 21	162 ± 5*
	NGFR	100 ± 3	52 ± 4*	76 ± 8	46 ± 0.2^††^
	CamK2a	100 ± 5	61 ± 4*	85 ± 3	104 ± 8
	CamK2d	100 ± 9	137 ± 2*	117 ± 11	116 ± 10
Interferons	IFNα	100 ± 5	153 ± 5*	200 ± 23**	302 ± 1^††^
	IFNϒ	100 ± 9	160 ± 8**	39 ± 1**	94 ± 3

*p < 0.05, **p < 0.01 vs control; †*p* < 0.05, ††*p* < 0.01 vs ethanol

### Microglia successfully repopulate in brain slice culture after PLX3397 removal

**Fig. 5:**
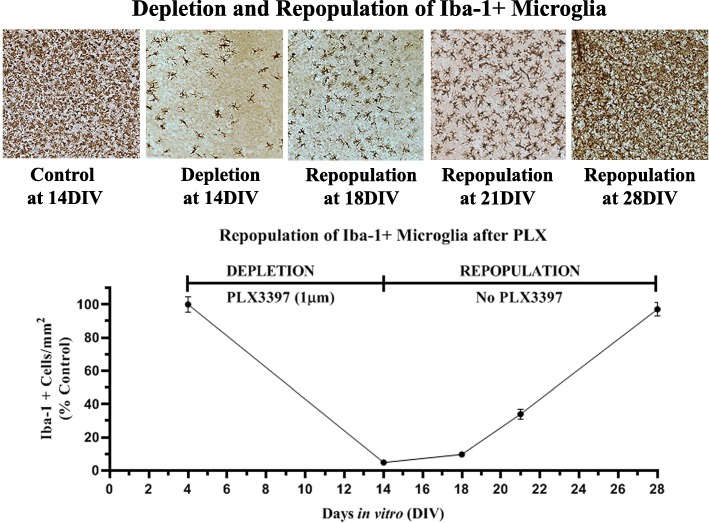
Microglia repopulate following removal of PLX3397. Iba-1 immunohistochemistry showing that Iba-1+ microglia were depleted after 10 days of treatment of PLX3397, with subsequent repopulation across 14 days after PLX3397 cessation. Quantification of Iba-1 microglia found greater than 95% depletion of microglia which were repopulated progressively back to control levels after 14 days

**Fig. 6:**
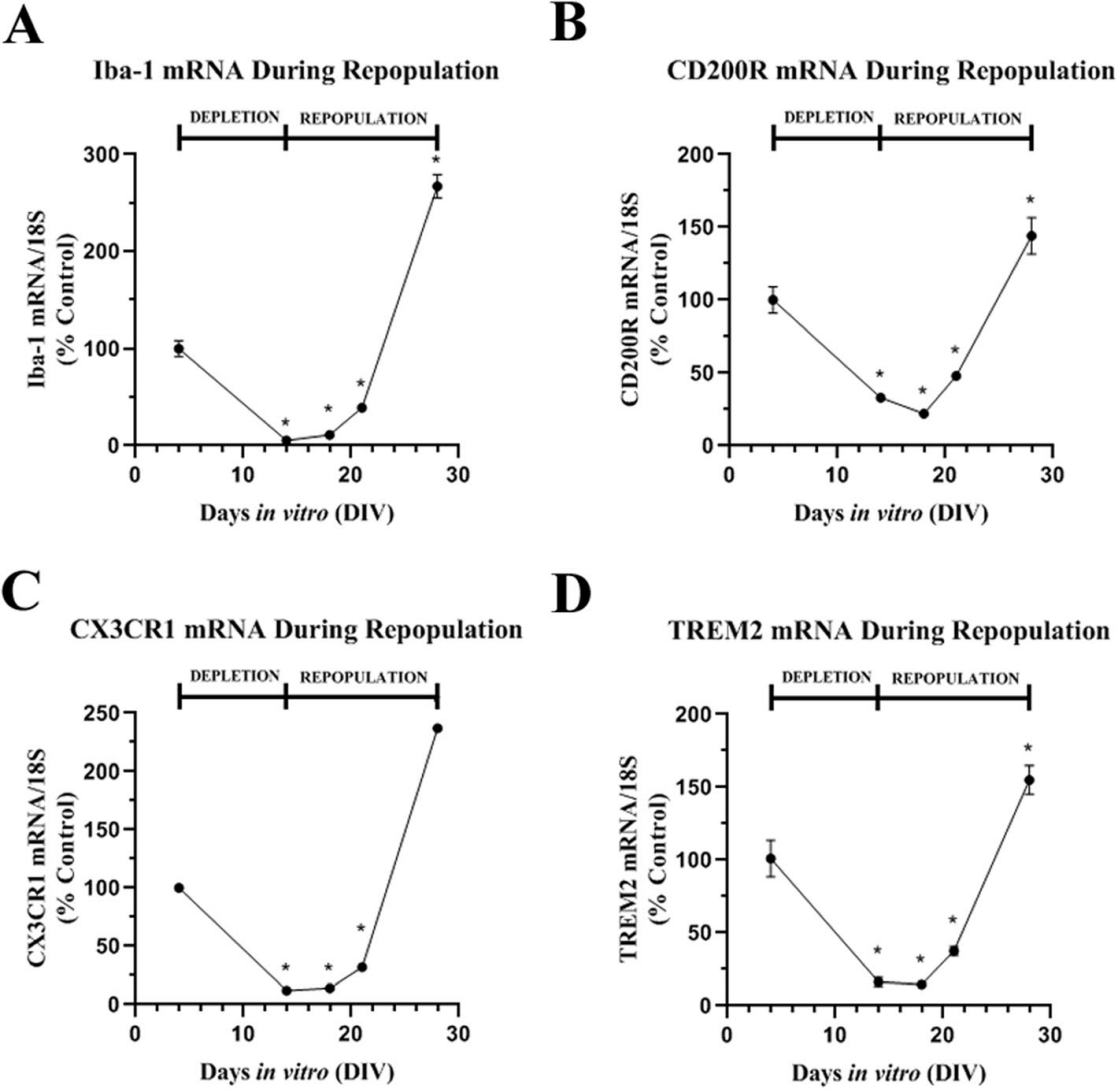
Restoration of microglia markers in new repopulated microglia. Common markers of microglia were assessed during microglial depletion and repopulation by RT-PCR. Microglial depletion caused a reduction during PLX3397 treatment (4DIV-14DIV), followed by a restoration after PLX3397 was removed (14DIV-28DIV) of the markers **a** Iba-1, **b** CD200R, **c** CX3CR1, and **d** TREM2. *N* = 6–8 replicates/group. *p < 0.05 vs control matched for days in vitro

Many studies have found depletion of microglia followed by repopulation has beneficial effects on neuropathology [17]. Therefore, we investigated whether microglia would repopulate in the OHSC after PLX3397 depletion. Slices exposed to PLX3397 for 10 days to deplete microglia and were followed for an additional 14 days (14DIV) without PLX3397 (Fig. 1b). Microglial number was assessed after depletion using Iba-1+ IHC (Fig. 5). PLX3397 treatment (4–14DIV) reduced Iba-1+ cells more than 90% at DIV14. Removal of PLX3397 by 18DIV showed a trend that by 21DIV had recovered microglial Iba-1+ cells to 30% of control levels. After 14 days without PLX3397, Iba-1+ microglia repopulated to original levels at 28DIV. Pretreatment with BrdU to label dividing cells during repopulation found Iba-1 co-localized with BrdU, confirming repopulation of newly formed microglia by proliferation (Additional file 5: Figure S3). The return of microglia was associated with a progressive increase in gene expression of microglial genes Iba-1, CD200R, CX3CR1, and TREM2 (Fig. 6). The expression of these genes surpassed baseline levels even though the total number of repopulated microglia returned to pre-treatment levels.

**Fig. 7:**
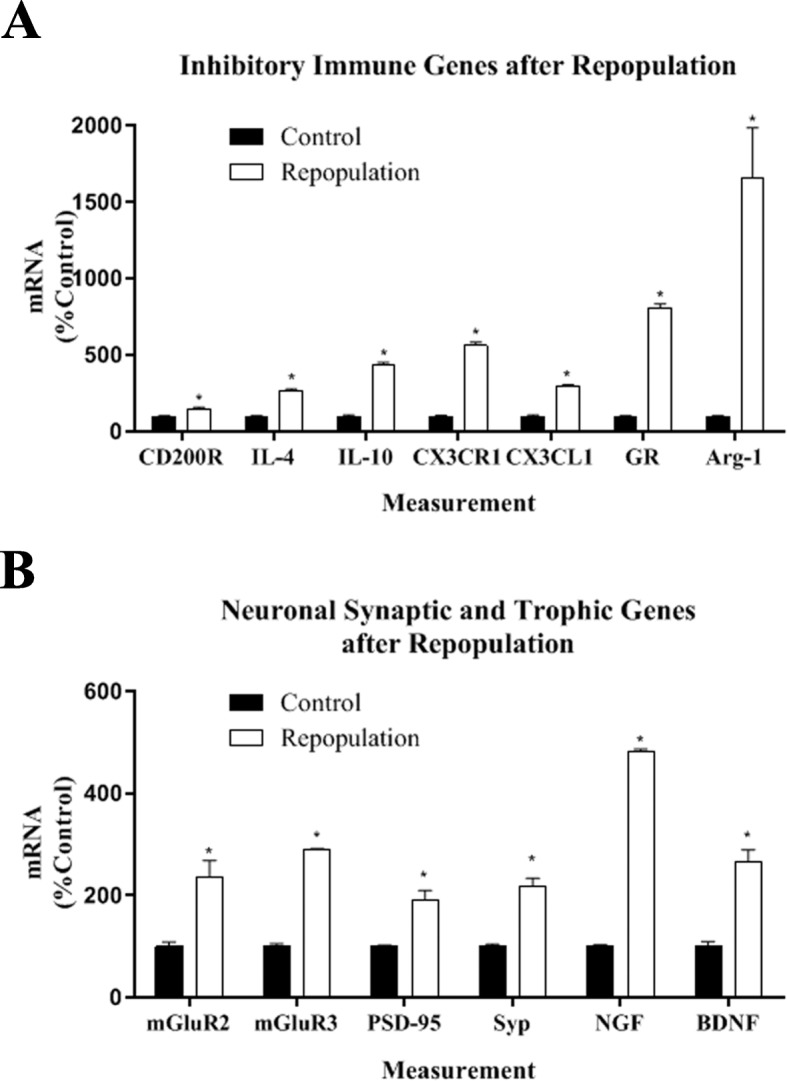
Microglial repopulation increases inhibitory immune signals, neuronal synaptic and trophic genes. OHSCs were treated with PLX3397 for 10 days in vitro (DIV) to deplete microglia and followed by microglial repopulation in PLX3397-free medium for different time points. Gene expression was measured by RT-PCR. **a** Microglial repopulation was associated with an increased induction of immune inhibitory and M2-anti-inflammatory genes CD200R (1.4-fold), IL-4 (2.7-fold), IL-10 (4.4-fold), CX3CR1 (5.6-fold), CX3CL1 (3-fold), GR/glucocorticoid receptor (8-fold), and Arg-1 (16.6-fold). **b** Microglial repopulation caused increased expression of neuronal synaptic and trophic genes mGluR2 (2.4-fold), mGluR3 (2.9-fold), PSD-95 (1.9-fold), synaptophysin/Syp (2.2-fold), NGF (4.8-fold), and BDNF (2.7-fold). *p < 0.05 vs control matched for days in vitro

We next investigated whether repopulation of microglia altered the expression of cytokines, TLRs, microglia inhibitory signals, neuronal synaptic markers, and trophic genes (Table 3). Pro-inflammatory cytokines (or traditionally M1 genes) such as TNFα and IL-1β returned to near normal levels. However, microglial inhibitory (traditionally M2) and anti-inflammatory cytokines IL4 and IL10 showed increased expression with repopulated microglia. Microglial receptors CD200R and CX3CR1 were increased after microglial repopulation (1.4- and 5.6-fold respectively, Fig. 7a). Increases in anti-inflammatory genes IL-10 (4.4-fold) and glucocorticoid receptor (GR, 8-fold), with the large increase in the microglial fractalkine receptor (CX3CR1, 5-fold) and neuronal fractalkine (CXCL1-3 fold), are all consistent with OHSC with repopulated microglia having a more “anti-inflammatory” phenotype (Fig. 7). Repopulated microglia had increased expression of the traditional M2 marker Arg-1 (16.6-fold) and phagocytic marker CD68 (7.8-fold). In addition, OHSC with repopulated microglia had increased expression of tropic factor genes NGF (4.8-fold) and BDNF (2.7-fold) (Fig. 7b). Interestingly, there was an increase in neuronal synaptic genes (mGluR2, mGluR3, PSD-95, synaptophysin/syp).

**Table 3:** Repopulation of microglia changes immune and neuronal gene expression. Brain slices were treated with vehicle or underwent microglial depletion with PLX3397 for 10 days, followed by repopulation for zero, 7 or 14 days. Gene induction was assessed by RT-PCR. Mean ± SEM

Category	Gene	Control	Repopulation day Zero	Repopulation day 7	Repopulation day 14
Cytokines	TNFα	100 ± 12	22 ± 4**	65 ± 6**	104 ± 8
	IL-1β	100 ± 2	15 ± 2**	24 ± 1**	87 ± 6
	IL-4	100 ± 14	211 ± 2**	303 ± 58**	267 ± 10**
	IL-6	100 ± 14	93 ± 6	98 ± 1*	202 ± 5**
	IL-10	100 ± 4	92 ± 6	163 ± 11*	436 ± 14**
Toll-like receptors	TLR2	100 ± 0.4	15 ± 2*	22 ± 1*	131 ± 16*
	TLR3	100 ± 2	17 ± 0.5*	19 ± 1*	164 ± 9*
	TLR4	100 ± 3	40 ± 4*	51 ± 1*	241 ± 19*
	TLR5	100 ± 5	20 ± 1*	11 ± 2*	364 ± 28*
	TLR7	100 ± 9	19 ± 1*	8 ± 0.3*	182 ± 6*
	TLR9	100 ± 8	12 ± 1**	10 ± 0.3*	194 ± 11*
Microglia markers	Iba-1	100 ± 0.3	16 ± 1.3*	16 ± 0.4*	238 ± 49*
	TREM2	100 ± 9	16 ± 1*	9 ± 0.3*	267 ± 11*
	CD200R	100 ± 9	33 ± 1**	48 ± 4*	144 ± 13**
	CX3CR1	100 ± 3	15 ± 2*	8 ± 0.2*	564 ± 18*
	IL-4R	100 ± 10	41 ± 2*	98 ± 1*	202 ± 5*
	IRF8	100 ± 2	16 ± 0.5*	8 ± 0.1**	160 ± 9*
	CD68	102 ± 19	48 ± 1*	22 ± 0.2*	776 ± 94**
	C1qA	100 ± 10	8 ± 1*	13 ± 0.4**	113 ± 4
	C3	100 ± 12	6 ± 0.6*	7 ± 0.1**	47 ± 7**
Neuronal genes	mGluR2	100 ± 8	126 ± 10*	332 ± 26**	237 ± 31**
	mGluR3	101 ± 4	146 ± 12*	142 ± 1*	289 ± 2**
	mGluR5	100 ± 8	131 ± 4*	163 ± 9**	404 ± 34**
	mGluR8	100 ± 4	227 ± 30*	176 ± 2**	677 ± 44**
	NR2A	100 ± 3	143 ± 5*	165 ± 15*	212 ± 17**
	NR2B	100 ± 5	127 ± 8*	173 ± 7*	304 ± 26**
	PSD-95	100 ± 2	168 ± 11*	134 ± 12*	191 ± 18**
	Synapsin	100 ± 4	152 ± 4*	116 ± 2	219 ± 14**
	CX3CL1	100 ± 8	98 ± 2	79 ± 9*	295 ± 10**
Other genes	Arg-1	100 ± 3	165 ± 28*	144 ± 22*	1655 ± 328**
	GFAP	100 ± 2	90 ± 1	133 ± 12*	148 ± 0.5*
	GR	100 ± 3	97 ± 2	72 ± 5*	806 ± 28**
	BDNF	100 ± 9	105 ± 6	135 ± 8*	265 ± 24**
	NGF	100 ± 3	186 ± 9*	153 ± 10*	481 ± 5*

*p < 0.05, **p < 0.01 vs control

**Fig. 8:**
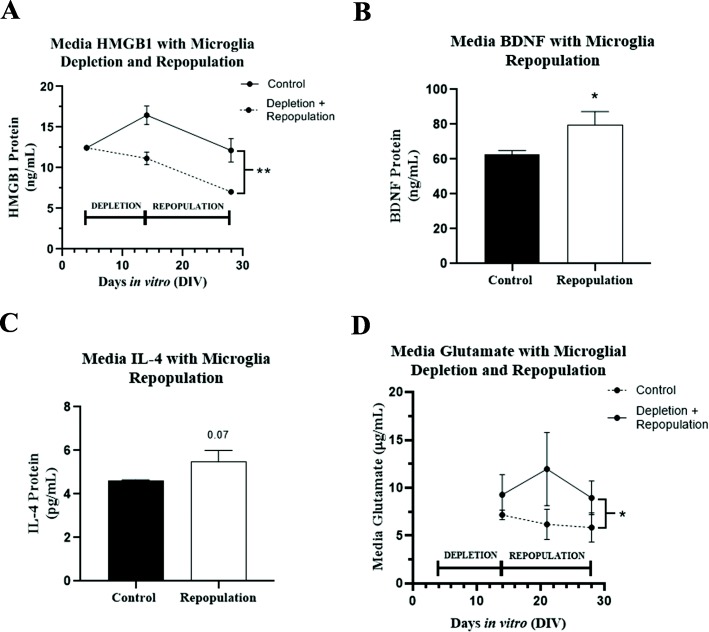
Media cytokine and immune proteins match slice mRNA changes. OHSCs were treated with PLX3397 for 10 days in vitro (DIV) beginning on DIV4 to deplete microglia, followed by microglial repopulation in PLX3397-free medium for different durations. Media protein levels for glutamate, HMGB1, BDNF, and IL-4 were measured by ELISA. **a** Protein levels of the proinflammatory mediator HMGB1 was assessed by ELISA prior to depletion (DIV4), after depletion (DIV14) and after repopulation (DIV28). There was a significant treatment effect of microglial depletion and repopulation on media HMGB1 levels compared to untreated age-matched control cultures F(1,5) = 22.66, **p < 0.006. **b** Protein levels of the trophic Brain-derived neurotrophic factor (BDNF) were measured in culture media by ELISA after microglial repopulation. BDNF was increased by 28% in cultures that underwent microglial depletion and repopulation compared to age-matched untreated controls *p < 0.05, *t* test. **c** Media IL-4 protein was measured by ELISA after microglia repopulation. IL-4 showed a trend toward a significant increase after microglia repopulation (*p =* 0.07, *t* test). **d** OHSCs were grown in glutamate-free media, supplemented with l-glutamine (2 mM). Media glutamate was measured in OHSC media immediately after depletion (DIV14) and on repopulation day 14 (DIV 21) and 21 (DIV 28). Two separate experiments were analyzed at each time point. A transient increase in media glutamate was observed in the microglial depletion/repopulation group. ANOVA: F_1,3_ = 10.5, *p < 0.05

To determine if these changes in gene expression corresponded with changes in secreted pro-inflammatory and trophic proteins, we measured media HMGB1 (pro-inflammatory TLR4 agonist), brain-derived neurotrophic factor (BDNF), and IL-4 from OHSCs that underwent microglia depletion and repopulation compared to age-matched untreated controls. Media HMGB1, an endogenous TLR4 agonist, was significantly reduced in media from OHSCs that underwent microglial depletion and repopulation (Fig. 8a, main effect of treatment F(1,5) = 22.66, **p < 0.006). Media BDNF was significantly increased (Fig. 8b, 27% increase, *p <* 0.05) consistent with slice mRNA assessments. Media IL-4 showed a trend toward an increase (Fig. 8c, *p =* 0.07) similar to slice mRNA assessments. Thus, these findings support that repopulation of microglia after PLX3397 results in a more anti-inflammatory, trophic environment with increases in expression of synaptic neuronal, microglial anti-inflammatory, and trophic genes. This was accompanied by a moderate transient increase in media glutamate during repopulation that returned to near control levels by day 14 of repopulation (DIV28 overall, Fig. 8d, F(1,3) = 10.2, *p < 0.05). Whether this is due to neuronal release or changes in astrocyte reuptake or release will be assessed in future studies.

### Repopulated microglia have blunted responses to LPS, IMQ, and binge ethanol

**Fig. 9:**
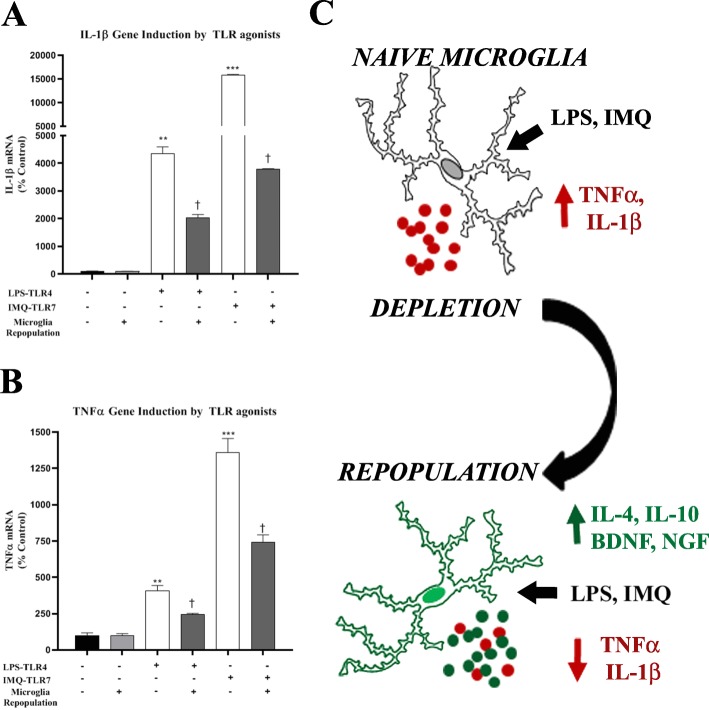
Microglial repopulation blunts proinflammatory responses to TLR4 and TLR7 agonists. Ex vivo OHSCs underwent microglial depletion followed by repopulation. On day 14 of repopulation, slices were treated with agonists to TLR4 (LPS 100 ng/ml) and TLR7 (Imiquimod, IMQ 5 μg/ml) for 16 h. Induction of proinflammatory genes TNFα and IL-1β was assessed by RT-PCR. **a** IL-1β was strongly induced in naïve cultures (28DIV) by LPS (44-fold), and IMQ (158-fold). Microglia-repopulated slices showed reduced induction of TNFα due to LPS (20-fold vs control, 55% reduction) and IMQ (38-fold vs control, 76% reduction). **b** TNFα was induced in naïve cultures (28DIV) by LPS (4-fold), and IMQ (13.6-fold). Microglia-repopulated slices showed reduced induction of TNFα due to LPS (2.5-fold vs control, 40% reduction) and IMQ (7.4-fold vs control, 45% reduction). **p < 0.01, ***p < 0.001 vs control. †*p <* 0.05 vs LPS or IMQ alone. *N* = 3 replicates/group, two independent experiments per condition. **c** Illustration depicting changes in immune gene expression in response to TLR agonists after microglial repopulation

**Fig. 10:**
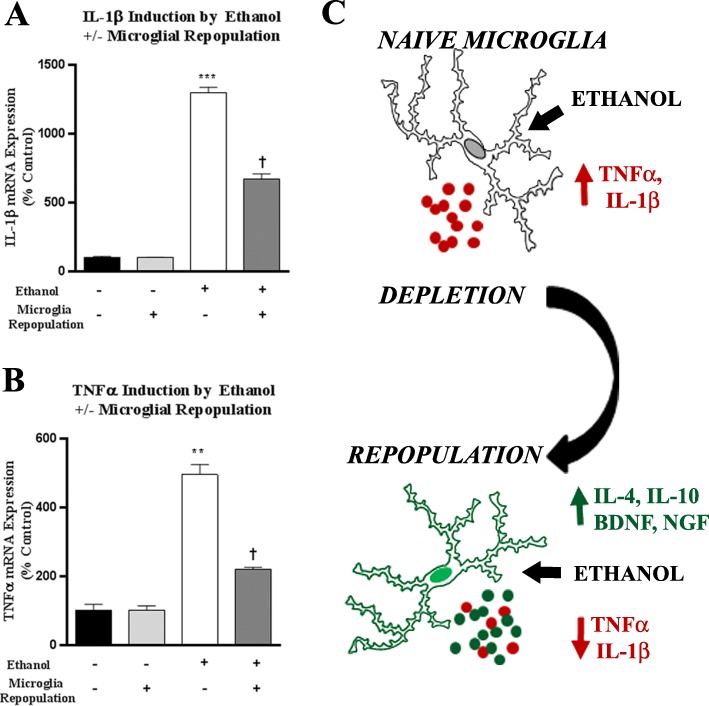
Microglial repopulation reduces proinflammatory responses to ethanol. OHSCs after microglial depletion and repopulation were treated with ethanol (100 mM) for 4 days and then followed by RT-PCR analysis. **a** Ethanol caused a 13-fold induction of IL-1β in naïve 28DIV slices. Microglia-repopulated slices showed reduced induction of IL-1β (6.4-fold vs control, 48% reduction). **b** Ethanol caused a 5-fold induction of TNFα in naïve 28DIV slices. Microglia-repopulated slices showed reduced induction of TNFα (2.2-fold vs control, 55% reduction). **p < 0.01, ***p < 0.001 vs control, †*p <* 0.05 vs ethanol. **c** Illustration depicting changes in immune gene expression in response to ethanol after microglial repopulation

Since we found successful repopulation of microglia in brain slice culture after depletion, with signs of an anti-inflammatory, trophic environment, we next assessed responses to immune activation. OHSC underwent microglial depletion and repopulation, and then were stimulated with either LPS (TLR4 agonist) or IMQ (TLR7 agonist) (Fig. 1b). All treated slices were compared to age-matched controls (i.e., 28DIV) that did not undergo PLX3397-microglial depletion and repopulation. Both LPS and IMQ induced TNFα and IL-1β in 28DIV naïve slices without depletion and repopulation (Fig. 9a, b). However, repopulated microglial slices showed significantly reduced TNFα and IL-1β responses. LPS-TLR4 induction of TNFα and IL-1β was reduced by 50%, and IMQ-TLR7 induction of TNFα and IL-1β were reduced by 40 and 77%, respectively. The reduction of proinflammatory TLR responses in microglia-repopulated slices is consistent with alterations in repopulated microglial phenotypes and the observed increased baseline levels of IL-10, CD200R, CX3CR1, BDNF, and NGF following repopulation (Fig. 7a, b). As ethanol is a biological exposure that activates TLRs, we next assessed responses to ethanol after microglial repopulation. Binge ethanol caused a 5-fold induction of TNFα and a 13-fold induction of IL-1β in naïve slices (28DIV, Fig. 10a, b). The induction of TNFα and IL-1β by binge ethanol was reduced by 60% in slices that underwent microglial depletion and repopulation. Thus, microglial depletion and repopulation results in reduced pro-inflammatory responses to TLR agonists and chronic ethanol. This may be related to an increase in microglial inhibitory signaling, anti-inflammatory cytokines, and trophic factors seen after microglial repopulation (Fig. 10c).

### Microglia repopulation normalizes persistent proinflammatory gene induction by chronic binge ethanol

**Fig. 11:**
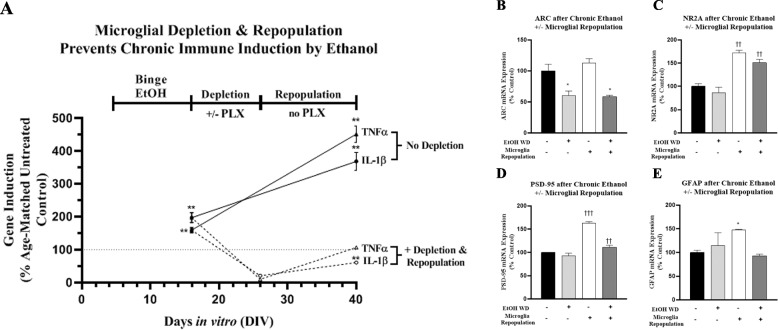
Microglial repopulation normalizes persistent proinflammatory responses to chronic binge ethanol. Primary ex vivo organotypic hippocampal slice cultures (OHSCs) underwent a model of chronic binge ethanol. OHSCs at 4DIV were exposed to ethanol (100 mM) for 3 cycles of 2 days on/ 2 days without ethanol. At the end of binge ethanol exposure (total duration 12 days), slices received either PLX3397 or vehicle for 10 days (leading to microglial depletion) followed by microglial repopulation for 14 days without PLX3397 or ethanol. Naïve control groups remained in culture without PLX3397 or ethanol for the same duration. **a** Chronic binge ethanol (4DIV-16DIV) caused a persistent increase in expression of TNFα (3.7-fold) and IL-1β (4.5-fold) out to 40DIV. Microglial repopulation normalized proinflammatory gene expression. Slices that underwent chronic ethanol followed by microglial repopulation showed expression of TNFα (107% of control) and IL-1β (61% of control) near age-matched control levels (naïve 40DIV slices). **b**–**e** Effects of microglial repopulation on neuronal **b** ARC, **c** NR2A and **d** PSD-95 and the astrocyte marker **e** GFAP

Microglial priming, or increased sensitization to proinflammatory signaling, has been implicated in the pathology of Alzheimer’s, Parkinson’s, AUD and other diseases. Microglial priming of proinflammatory signals can be induced by systemic LPS or binge ethanol exposure [5, 62–65]. Our previous in vivo findings indicate proinflammatory genes and microglial priming persist for long periods [66–68]. To investigate microglial sensitization and repopulation, we utilized a model of cycles of binge ethanol intoxication (2 days on/2 days off), as we have done previously in vivo and found increased brain proinflammatory gene expression [69, 70]. We then assessed whether microglial depletion and repopulation would protect against persistent ethanol effects (Fig. 1c). Importantly, OHSC expression of TNFα and IL1β was stable for long periods of culture in untreated controls (i.e., DIV14 to DIV42, Additional file 2: Table S2). In contrast, OHSC exposed to chronic binge ethanol showed approximately 2-fold increases in mRNA levels of TNFα and IL1β. This increased further over time to approximate 4-fold increases 24 days after ethanol exposure ended (Fig. 11a). This is consistent with in vivo studies finding persistent induction of neuroimmune genes after ethanol exposure as well as in postmortem human alcoholic brain tissue [9, 41]. Microglial depletion and repopulation after chronic binge ethanol prevented the progressive increase in TNFα and IL1β resulting in expression levels equal to untreated controls at 40DIV (Fig. 11a).

**Fig. 12:**
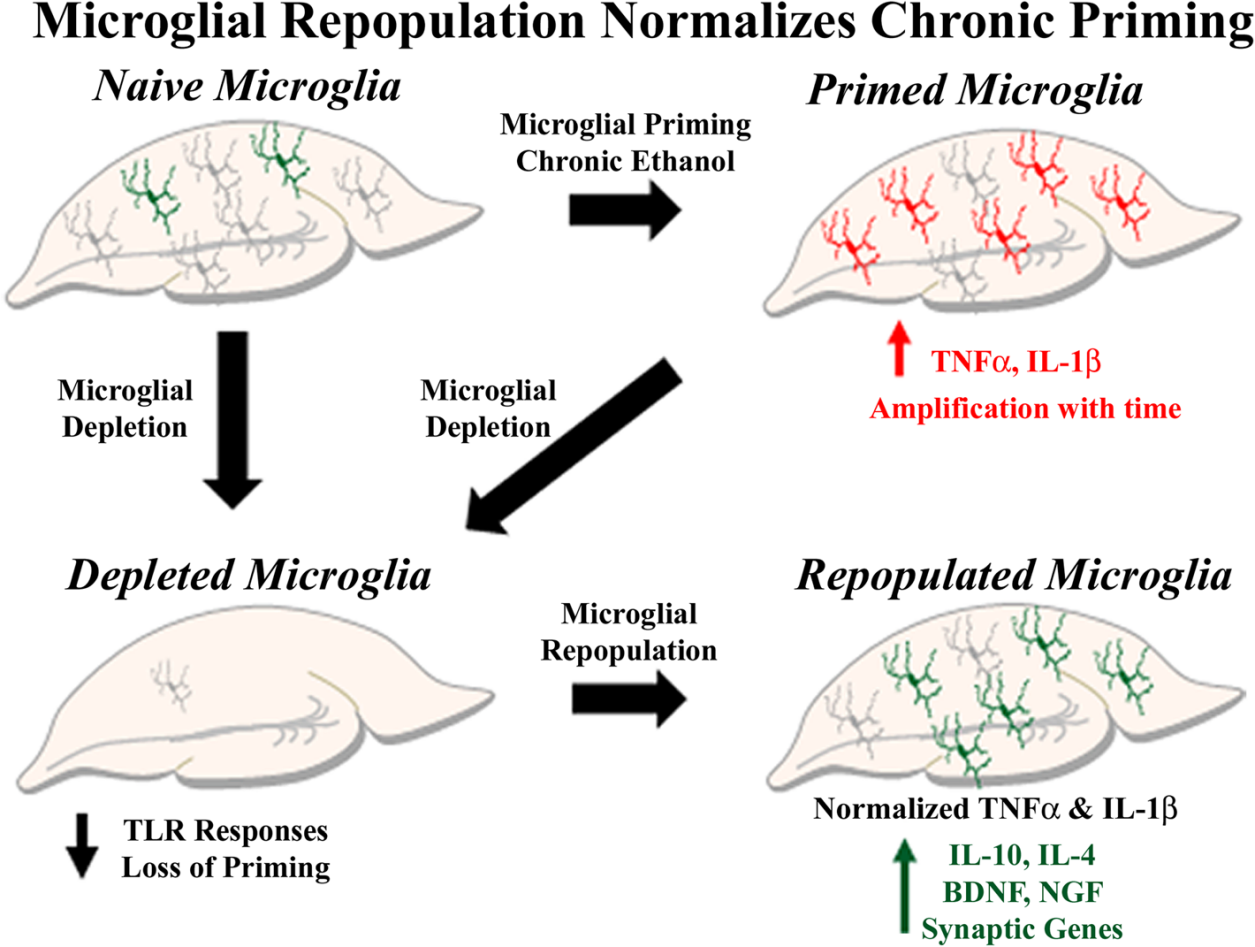
Summary of the beneficial effects of microglial repopulation on neuroimmune signaling. Microglial depletion was associated with reduced immune responses to both TLR agonists and ethanol. Chronic ethanol causes a long-lasting increase in proinflammatory cytokines consistent with innate immune priming. Microglial depletion with repopulation after ethanol returned innate immune gene expression to baseline levels

Microglial repopulation had modest and varying effects on neurons and astrocytes during protracted withdrawal after chronic binge ethanol. Chronic binge ethanol caused a persistent reduction in ARC neuronal immediate-early gene expression that was not restored by microglial repopulation (Fig. 11b). The mature neuronal glutamatergic NR2A subunit was minimally affected by chronic ethanol, though microglial repopulation caused a 70% increase in its gene expression, even after chronic ethanol (Fig. 11c). Chronic ethanol caused a slight reduction in the post-synaptic synapse gene PSD-95 immediately after ethanol (DIV14, 15%, *p < 0.05, not shown) that returned to control levels during withdrawal (Fig. 11d). Repopulation caused a 50% increase in PSD-95 and minimally increased its expression after chronic ethanol. Regarding astrocytes, chronic binge ethanol had no long-term effect on GFAP expression (Fig. 11e). Thus, microglial repopulation restores basal microglial phenotypes, thereby reversing proinflammatory microglial priming. These and previous findings are summarized graphically, showing the ability of microglial repopulation to reverse chronic pro-inflammatory activation and enhance trophic and anti-inflammatory signaling (Fig. 12).

## Discussion

We report here that OHSC microglia are depleted by PLX3397 and are largely responsible for TLR agonist and ethanol induction of proinflammatory signaling molecules. OHSC TLR proinflammatory signaling does not have the confounders of systemic TLR signals and/or the potential for peripheral immune cell migration as may occur in vivo [7, 23, 27]. TLR 2-9 mRNA was reduced 50–80% by microglial depletion. Endogenous agonists can activate TLR receptors in brain, and ethanol has been reported to activate brain HMGB1-TLR4 [39, 59] as well as Let7-TLR7 [43] signaling in brain. Microglial depletion reduced baseline expression of TNFα and IL1β and responses to TLR agonists and ethanol induction of these proinflammatory cytokines, supporting their role as key responders to these insults in brain. Our finding of reduced proinflammatory responses with microglial depletion is consistent with in vivo studies finding microglial depletion is protective in models of intracerebral hemorrhage [12] and diphtheria-induced hippocampal degeneration [13]. However, microglial responses are complex, and they seem to be critical first responders in settings such as ischemic stroke, where microglial depletion is detrimental [22, 23].

In contrast to the dramatic TLR-stimulation of TNFα and IL1β mRNA (10- to 275-fold), TLR induction of IFNs was lower at this time point (2- to 4-fold). Microglial depletion caused slight increases in baseline mRNA expression of IFN genes, but blunted responses to TLRs 2, 3, 4, and 9 agonists, although the TLR7-induction of IFNα was slightly enhanced. Similar to TLR7, ethanol induction of IFNα was not reduced by depletion, but was moderately enhanced. Type 1 IFNs, like IFNα, are known to impact microglial repopulation and phenotype [7] and degeneration [71] in vivo, as well as the risk of depression [72–76]. IFNγ is a type II interferon known for activating macrophages and other leukocytes. Interestingly, IFNγ mRNA was significantly increased by microglial depletion, which was further increased by agonists to TLRs 3, 4, and 9. Neuronal fractalkine (CX3CL1) conveys anti-inflammatory signals to microglia through CX3CR1, which was greatly reduced by microglial depletion. TLR2, TLR4, and TLR9 all increased CX3CL1, which for TLR2 and 4 agonists was more than doubled in the setting of microglial depletion, suggesting this enhancement of neuronal CX3CL1 is regulated in part by microglial feedback in these instances. CX3CR1 KO mice have transient developmental reductions in microglia number and concomitant impairment of synaptic pruning resulting in increased dendritic spines and immature synapses [77]. Similarly, we found microglial depletion caused increased expression of glutamate receptor subunits as well as PSD-95 mRNA, a marker of excitatory synapses. Expression of CD200, a neuronal and endothelial signal to CD200R on microglia that reduces TLR responses in mice, was different from CX3CL1/CXCR1 signaling [78, 79]. TLR3 decreased CD200 mRNA, and across other groups, it was either unchanged or slightly decreased, suggesting microglial-TLR responses do not enhance CD200-CD200R expression. Together, these findings are consistent with microglial-TLR signaling inducing TNFα and ILβ proinflammatory cytokines, with differential effects on induction of IFNs and neuronal-microglial regulatory signals.

Previous studies have used genetic and/or pharmacological strategies with clodronate liposomes in slice cultures to deplete microglia followed by microglia replenishment with adoptive transfers [37, 38, 80]. In these cases, microglia adopt an in vivo-like distribution with typical ramified morphology. We find that microglial repopulation fully occurs in ex vivo OHSC. This is consistent with observations in vivo [24, 25, 81–83]*.* To our knowledge, this is the first time PLX3397 has been used in OHSC to deplete microglia and study microglial their restoration. OHSCs with repopulated microglia had increased expression of multiple microglial mRNA including Iba-1, CD200R, CXCR1, arginase 1, IL10, IL4, and GR. Increased expression of GR could impact microglial proinflammatory responses and hypothalamic-pituitary axis function. Microglia (Iba1+) cell numbers returned to control levels after repopulation. Gene expression comparisons were made to untreated controls (i.e., without depletion) that spent the same number of days in culture. Importantly, the gene expression of TNFα and IL-1β did not change with days in culture (Additional file 2: Table S2). Thus, the microglial phenotypes change with repopulation, though the cell numbers are unchanged. Further, we found increased expression of trophic factors, BDNF, and NGF as well as glutamate receptors that could be expressed on repopulated microglia or on other cells in response to microglial repopulation. These genes suggests the OHSCs with repopulated microglia have a more “trophic anti-inflammatory” phenotype, with increased expression of traditional M2 markers, though TREM2 mRNA, which is linked to TLR signaling and phagocytosis, was increased in microglia-repopulated OHSCs. Further, we observed blunted TLR4- and TLR7 agonist-induced proinflammatory cytokine responses in repopulated microglia, consistent with an altered microglial phenotype. In previous reports in mice, microglia repopulation did not result in different responses to low doses of systemic LPS (0.25 mg/kg and 0.33 mg/kg in young and aged mice respectively) [21, 84]. LPS does not normally cross the blood-brain barrier in vivo, rather LPS-induced cytokines such as TNFα enter into brain to activate microglia [68], whereas slice tissue was directly exposed to LPS in our OHSC model to directly activate TLR4. Thus, it is possible that protection by microglial repopulation in vivo might be seen at higher concentrations of systemic LPS or with other clinical settings involving robust TLR activation in brain. Nevertheless, microglia repopulation in vivo results in restoration of age-associated microglial morphology changes [85] and normalizes many baseline age-related inflammatory gene changes in microglia [84] similar to our findings. Further, reports suggest that in vivo microglia repopulate from both F4/80^hi^Clec12a+ peripheral macrophages and remaining F4/80^low^Clec12a– microglia, with F4/80^hi^ displaying a more proinflammatory phenotype [7]. Thus, in vivo responses of repopulated microglia may be heterogeneous depending on their origin. The OHSC model does not include the contribution of peripheral monocytes and suggest repopulated microglia of central origin may express a more anti-inflammatory phenotype.

Postmortem studies find AUD brain has elevated levels of expression of TLR and proinflammatory cytokines, altered microglia, and neurodegeneration. Ethanol induces proinflammatory responses in brain, in part related to release of endogenous HMGB1 and Let-7, agonists at TLR4 and TLR7, respectively [39, 43, 59]. We found previously that ethanol administration alters brain microglia, and microglial depletion in mice blunts acute binge ethanol withdrawal-induced increases in brain TNFα and IL-1β mRNA [16], consistent with our findings that microglial depletion and restoration in OHSC blunts proinflammatory ethanol and TLR-induced proinflammatory mRNA responses. Interestingly, we found ethanol exposure of OHSC induced a long-lasting, persistent increase in TNFα and IL-1β mRNA that doubled over time, perhaps modeling microglial proinflammatory priming seen in vivo. Further, we found microglial depletion markedly reduced these mRNAs, suggesting they were mostly microglial, and with repopulation they return to control levels, not the higher level of primed mRNA before depletion. These findings are consistent with microglial priming involving TLR and/or other proinflammatory activation of microglia and not a microglial phenotypic change due to an altered niche. These findings are consistent with new microglia from endogenous cells reversing sustained immune activation and enhancing brain trophic support. Thus, these findings are consistent with in vivo microglial depletion and repopulation studies showing a benefit in multiple disease models [17] as well as improvement of memory and normalization of synaptic marker expression in models of neurodegeneration [13, 86].

## Conclusions

In conclusion, these findings suggest microglial depletion and repopulation may be beneficial in the context of TLR-mediated neuroimmune activation. Newly born ex vivo microglia show reduced proinflammatory responses to TLR agonists. Repopulation results in a restoration of proinflammatory gene expression to baseline in a model of chronic neuroinflammation. Future studies will assess the ability of microglial repopulation to reverse neuroimmune activation and behavioral disruption in models of chronic ethanol in vivo.

## Supplementary information

**Additional file 1: Table S1.** Primers for RT-PCR analyses.

**Additional file 2: Table S2.** Cq values for TNFα and IL1β across Days in Vitro (DIV).

**Additional file 3: Figure S1.** Microglial Inhibition in ex vivo brain slice culture with Gi DREADD (hM4Di) blunts TLR7 and ethanol-mediated induction of TNFα. HEC slices were incubated with the AAV9.CD68.hM4Di-mCherry for 24 hours. a Immunofluorescent labeling of microglia with Iba-1 (green) and hM4Di-mCherry receptors (red) shows colocalization of Gi DREADD in Iba-1+ microglia b 24 h after DREADD transfection slices were treated with the TLR7 agonist IMQ (5 μg/mL, 16 h) +/- DREADD ligand CNO (0.5-1 μM). IMQ caused a 13-fold induction of TNFα. Inhibition of microglia with DREADD signaling blunted IMQ-induction of TNFα in a concentration-dependent manner, implicating microglia in this response. c 24 h after DREADD transfection slices were treated with the ethanol (100 mM) for 4 days +/- CNO (0.5-1 μM). Ethanol caused a 3.5-fold induction of TNFα. Inhibition of microglia with DREADD signaling blunted ethanol induction of TNFα in a concentration-dependent manner. ****p < 0.0001 vs control, ††*p* < 0.01, †††*p* < 0.001 vs IMQ or ethanol alone.

**Additional file 4: Figure S2.** Microglial depletion reduces induction and secretion of pro-inflammatory miRNAs by ethanol. OHSCs at 4DIV were treated with CSF1R inhibitor PLX3397 for 10 days to deplete microglia and followed by treatment of ethanol (100 mM, 4 days). Slices were removed for microRNA (miR) analysis and media microparticles were isolated for analysis of secreted miRNAs. a Ethanol induced the expression of let-7b, miR-155, miR-21, and miR-181c in slice tissue. Microglial depletion abolished the induction of miR-155, miR-21, and miR-181c. b Ethanol caused the secretion of let-7b, miR-155, miR-21, and miR-181c in media microparticles. Microglial depletion reduced the secretion of let-7b and miR-155, while reducing the ethanol-induced secretion of miR-21 miR-181c to below control levels. *p < 0.05 vs control; †*p* < 0.05 vs ethanol. N = 3 replicates/group.

**Additional file 5: Figure S3.** BrdU+ colocalizes with Iba-1 during repopulation of microglia. OHSCs at 4DIV were treated with PLX3397 (1uM) for 10 days to deplete microglia. BrdU was loaded in slices 24 hr before the end of PLX3397 treatment. Slices were returned to PLX3397-free, BrdU-free medium for different durations. Representative images show BrdU (red) and Iba-1 (green) immunofluorescence. At the end of microglial depletion (M-Dep), some BrdU+ cells were identified with few Iba-1+ microglia. As microglial repopulation occurred, the number of BrdU+ (red), Iba-1+ (green) and BrdU+/Iba-1+ cells (yellow) progressively increased.

## Data Availability

The datasets during and/or analyzed during the current study are included in this published article. Any additional data is available from the corresponding author on reasonable request.

## Abbreviations

AUD: Alcohol use disorder
BDNF: Brain-derived neurotrophic factor
CSF1R: Colony-stimulating factor 1 receptor
CX3CL1: Fractalkine
CX3CR1: Fractalkine receptor
DIV: Days in vitro
DREADD: Designer Receptor Exclusively Activated by a Designer Drug
hM4Di: Gi-inhibitory DREADD
IFN: Interferon
IMQ: Imiquimod
LPS: Lipopolysaccharide
MP: Microparticle
M-Dep: Microglial depletion
OHSC: Organotypic hippocampal slice culture
TLR: Toll-like receptor
