# Supplementary material for: Microglial depletion and repopulation in brain slice culture normalizes sensitized proinflammatory signaling
Source: J Neuroinflammation. 2020 Jan 18;17:27. doi: 10.1186/s12974-019-1678-y (PMC6969463; doi:10.1186/s12974-019-1678-y)
Supplement: Supplementary file 1 — Additional file 1: Table S1. Primers for RT-PCR analyses. [file 12974_2019_1678_MOESM1_ESM.docx]

| **Supplemental Table 1: Primers for RT-PCR analyses** | | |
| --- | --- | --- |
| **Genes** | **Forward (5’-3’)** | **Reverse (5’-3’)** |
| **TNFα** | AGCCCTGGTATGAGCCCATGTA | CCGGACTCCGTGATGTCTAAG |
| **IL-1β** | TTGTGCAAGTGTCTGAAGCA | TGTCAGCCTCAAAGAACAGG |
| **IL-4** | TGCACCGAGATGTTTGTACC | CGAGAACCCCAGACTTGTTC |
| **IL-6** | GGTCTTCTGGAGTTCCGTTT | GATGGTCTTGGTCCTTAGCC |
| **IL-10** | CAAAGGTGTCTACAAGGCCA | CAAGGAGTTGCTCCCGTTAG |
| **TLR2** | GGAGGTCTCCAGGTCAAATCT | TGAGTCCCGAGGGAATAGAG |
| **TLR3** | CTACAACAGCCTCCGCGAC | ACGAAAAGAGTGAGGGGTCA |
| **TLR4** | CAGGTCGAATTGTATCGCCT | GGTCGTTGAGGTTAGAAGCC |
| **TLR5** | ACCTCAAGCGTGTTCTCATC | ACCTCAAGCGTGTTCTCATC |
| **TLR7** | CAAGCTCTGTTCTCCTCCAC | AGTCTGGAGAGATGCTTGGT |
| **TLR8** | CTGAAGTTGTGGCCTATCCC | ACAGGATAGCTGCAGTGGTA |
| **TLR9** | GTTCGTCAACCTCTCCCATC | CTGAAGTTGTGGCCTATCCC |
| **IBa-1** | GAGCTATGAGCCAGAGCAAG | CCCCAAGTTTCTCCAGCATT |
| **TREM2** | AAGCTTCTTACAGCCAGCAT | GTAGCAGAACAGAAGTCTTGGT |
| **CD200R** | CTACAGTGTTTGTACAGATGGGT | AAATTCCCGTCAGGTACTGC |
| **CX3CR1** | CTTCTTCCTCTTCTGGACGC | CCTCGCTTGTGTAGTGAGTC |
| **IL-4R** | GAGCTGCATTCCAGTCTCTC | GTATGTGTCTGCCTGGATCG |
| **IRF8** | CTACAACCAGGAAGTGGACG | GAAGGCTCCTTGATCAGCTC |
| **mGluR2** | ACTCTGACATGAGGATTCCCT | TAGTATCCCACTCCCCACAC |
| **mGluR3** | AATGTGCATCTCCGTTAGCC | CTTGCAGAGGACTGAGAATAGG |
| **mGluR5** | CATGTTTGTCCCGAAGGTGT | TTTCCGTTGGAGCTTAGGG |
| **mGluR8** | TAATGGCAGTGCTGGTACAC | CGTGTGCTCTCTATTAGCCC |
| **NR2A** | GTGATCGTGCTGAACAAGGA | GCTCGCAGTCAGAAAAGGAC |
| **NR2B** | TCCGAAGCTGGTGATAATCC | TGGTCATCCTCTTGCTCCTC |
| **PSD-95** | CTACCAAGATGAAGACACGCC | GTTCCATTCACCTGCAACTC |
| **BDNF** | GTGACARTATTAGCGAGTGGG | GGGTAGTTCGGCA TTGC |
| **NGF** | CGCTCTCCTTCACAGAGTTTTG | TCAGAGTGGCCAGGATAGAA |
| **NGFR** | CTGCTGATTCTAGGGATGTCCT | GAATGTAACACTGTCCAGGCA |
| **CamK2a** | GGGAGCTGTTCGAAGACATT | TTCGAAGCCAGCAACAGATT |
| **CamK2d** | TGGCAAACTAAAGAGGGAGC | TCCCAATGAGAAGCCCAAATAG |
| **IFNα** | GTCTCATGCCTGAAGGACAG | TGAGTCTAGGAGGGTTGCAT |
| **IFNϒ** | CTGGCAAAAGGACGGTAACA | GTGATCAGGTGCGATTCGAT |
| **GR** | CCTTCTGGAGTGGTTTGCAT | AGTTGGTAAGGTGCACACAG |
| **C1qA** | AGCTTTCTCAGCTATTCGGC | GGAGGAGGACACGATAGACA |
| **β-Actin** | CTACAATGAGCTGCGTGTGGC | CAGGTCCAGACGCAGGATGGC |
