# Supplementary material for: Microglial depletion and repopulation in brain slice culture normalizes sensitized proinflammatory signaling
Source: J Neuroinflammation. 2020 Jan 18;17:27. doi: 10.1186/s12974-019-1678-y (PMC6969463; doi:10.1186/s12974-019-1678-y)
Supplement: Supplementary file 2 — Additional file 2: Table S2. Cq values for TNFα and IL1β across Days in Vitro (DIV). [file 12974_2019_1678_MOESM2_ESM.docx]

| **Supplemental Table 2:** Cq values for TNFα and IL-1β across Days in Vitro (DIV) | | |
| --- | --- | --- |
| **Treatment Group** | **TNFα Cq value** | **IL-1β Cq value** |
| **Control 14DIV** | **26.9** | **25.5** |
| **Control 21DIV** | **27.2** | **25.8** |
| **Control 35DIV** | **26.9** | **26.4** |
| **Control 42DIV** | **26.5** | **26.7** |
