# Supplementary figures and images for: Microglial depletion and repopulation in brain slice culture normalizes sensitized proinflammatory signaling
Source: J Neuroinflammation. 2020 Jan 18;17:27. doi: 10.1186/s12974-019-1678-y (PMC6969463; doi:10.1186/s12974-019-1678-y)

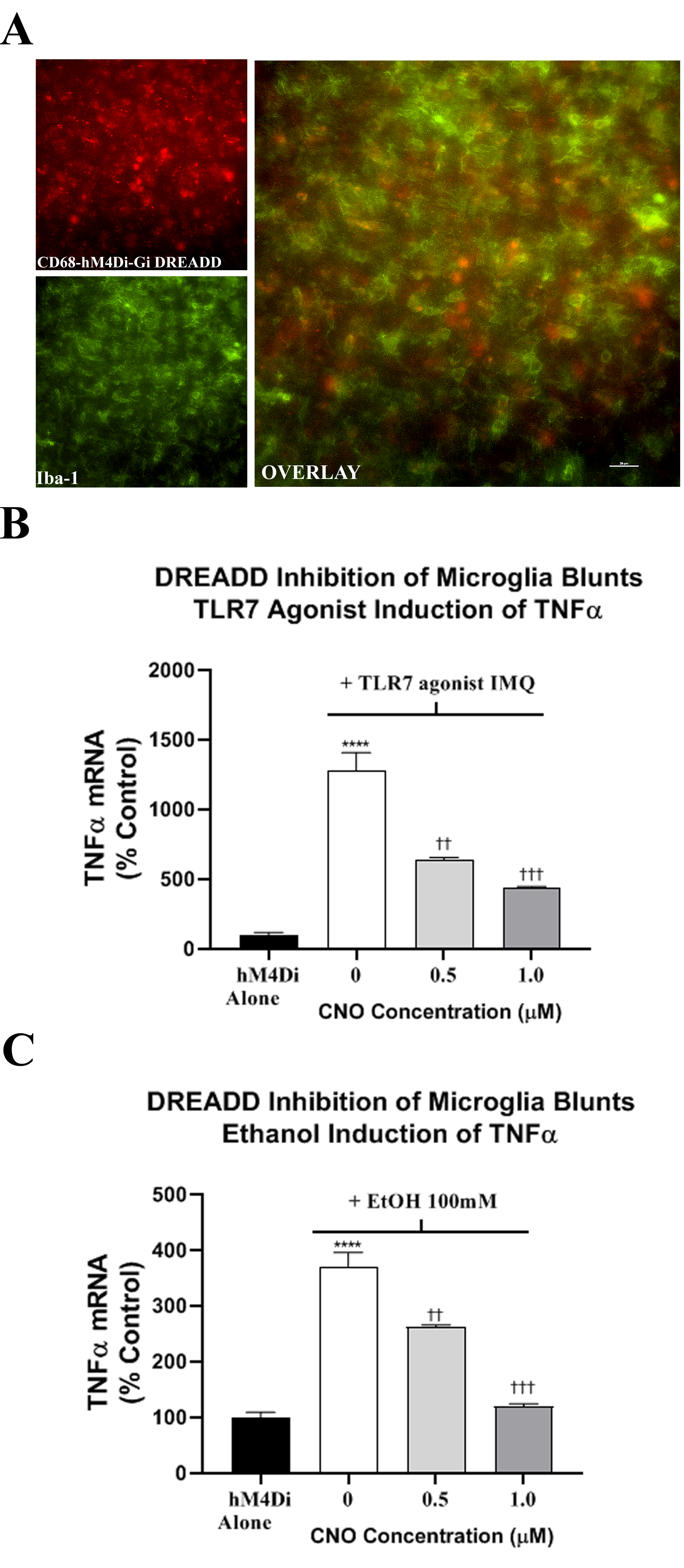

Supplement: Supplementary file 3 — Additional file 3: Figure S1. Microglial Inhibition in ex vivo brain slice culture with Gi DREADD (hM4Di) blunts TLR7 and ethanol-mediated induction of TNFα. HEC slices were incubated with the AAV9.CD68.hM4Di-mCherry for 24 hours. a Immunofluorescent labeling of microglia with Iba-1 (green) and hM4Di-mCherry receptors (red) shows colocalization of Gi DREADD in Iba-1+ microglia b 24 h after DREADD transfection slices were treated with the TLR7 agonist IMQ (5 μg/mL, 16 h) +/- DREADD ligand CNO (0.5-1 μM). IMQ caused a 13-fold induction of TNFα. Inhibition of microglia with DREADD signaling blunted IMQ-induction of TNFα in a concentration-dependent manner, implicating microglia in this response. c 24 h after DREADD transfection slices were treated with the ethanol (100 mM) for 4 days +/- CNO (0.5-1 μM). Ethanol caused a 3.5-fold induction of TNFα. Inhibition of microglia with DREADD signaling blunted ethanol induction of TNFα in a concentration-dependent manner. ****p < 0.0001 vs control, ††p < 0.01, †††p < 0.001 vs IMQ or ethanol alone. [file 12974_2019_1678_MOESM3_ESM.tif]

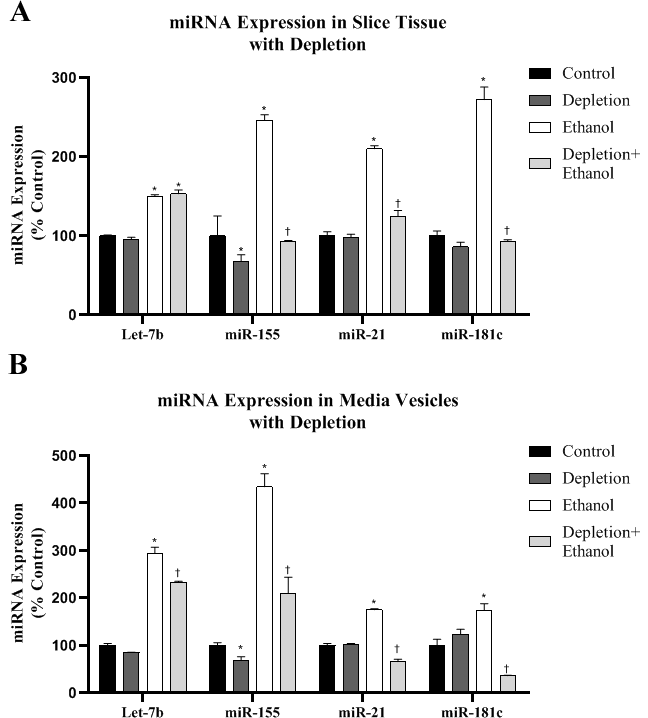

Supplement: Supplementary file 4 — Additional file 4: Figure S2. Microglial depletion reduces induction and secretion of pro-inflammatory miRNAs by ethanol. OHSCs at 4DIV were treated with CSF1R inhibitor PLX3397 for 10 days to deplete microglia and followed by treatment of ethanol (100 mM, 4 days). Slices were removed for microRNA (miR) analysis and media microparticles were isolated for analysis of secreted miRNAs. a Ethanol induced the expression of let-7b, miR-155, miR-21, and miR-181c in slice tissue. Microglial depletion abolished the induction of miR-155, miR-21, and miR-181c. b Ethanol caused the secretion of let-7b, miR-155, miR-21, and miR-181c in media microparticles. Microglial depletion reduced the secretion of let-7b and miR-155, while reducing the ethanol-induced secretion of miR-21 miR-181c to below control levels. *p < 0.05 vs control; †p < 0.05 vs ethanol. N = 3 replicates/group. [file 12974_2019_1678_MOESM4_ESM.tif]

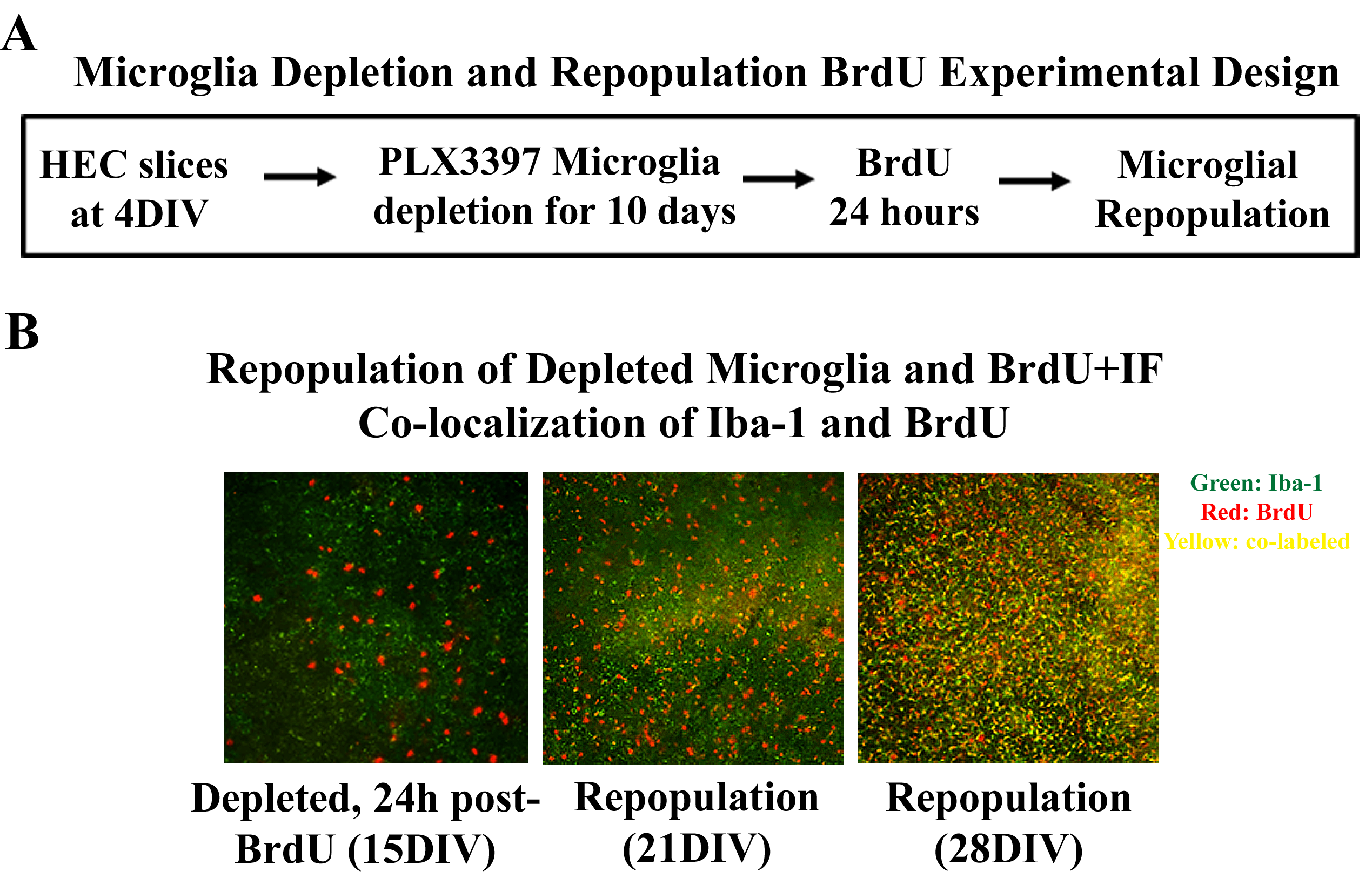

Supplement: Supplementary file 5 — Additional file 5: Figure S3. BrdU+ colocalizes with Iba-1 during repopulation of microglia. OHSCs at 4DIV were treated with PLX3397 (1uM) for 10 days to deplete microglia. BrdU was loaded in slices 24 hr before the end of PLX3397 treatment. Slices were returned to PLX3397-free, BrdU-free medium for different durations. Representative images show BrdU (red) and Iba-1 (green) immunofluorescence. At the end of microglial depletion (M-Dep), some BrdU+ cells were identified with few Iba-1+ microglia. As microglial repopulation occurred, the number of BrdU+ (red), Iba-1+ (green) and BrdU+/Iba-1+ cells (yellow) progressively increased. [file 12974_2019_1678_MOESM5_ESM.tif]
